# Coral Bleaching: The Equatorial‐Refugia Hypothesis

**DOI:** 10.1111/gcb.70594

**Published:** 2025-11-17

**Authors:** Zachary Ferris, Andrew S. Walker, Håvard Rue, Robert van Woesik

**Affiliations:** ^1^ Institute for Global Ecology Florida Institute of Technology Melbourne Florida USA; ^2^ Statistics Program, Computer, Electrical and Mathematical Sciences and Engineering Division King Abdullah University of Science and Technology Thuwal Saudi Arabia

**Keywords:** Bayesian inference, climate change, coral bleaching, coral reefs, marine heatwaves, ordered‐beta regression, refugia, spatially‐explicit statistics

## Abstract

The rising threat of marine heatwaves has led to numerous predictions that coral reefs, especially those near the Equator, will be severely degraded by the end of the current century. Yet, environmental conditions near the Equator may regionally moderate coral bleaching by reducing thermal stress during marine heatwaves. We deployed a Bayesian spatio‐temporal model over Earth to examine which environmental conditions may characterize marine‐heatwave refugia for coral reefs by testing the relationship between the severity of coral bleaching and a suite of temperature, hydrodynamic, topographic, atmospheric, and biological variables. The model considered the severity of coral bleaching as the proportion of bleached hard corals during 30,266 coral‐reef surveys conducted at 8728 sites, at depths of up to 20 m, and located between 35° north and south of the Equator across 81 countries, from 2002 to 2020. Except for the eastern Pacific Ocean, the severity of coral bleaching during marine heatwaves was lower on equatorial reefs than on higher‐latitude reefs, suggesting that marine‐heatwave refugia for corals have been concentrated in the equatorial Coral Triangle region. Indeed, equatorial reefs in the Coral Triangle were, on average, exposed to the weakest marine heatwaves, potentially because they were shielded from extreme insolation by frequent cloud coverage in the Intertropical Convergence Zone. Coral bleaching may also be moderated during marine heatwaves on reefs that experience high wave energy, high current velocity, high cloud frequency, or turbidity. Coral bleaching was also less severe on reefs that historically endured frequent heatwaves than on reefs that were naïve to thermal stress. Based on modern and historical responses of coral reefs to acute thermal stress, we hypothesize that many equatorial reefs will continue to serve as marine‐heatwave refugia for corals.

## Graphical abstract

Marine heatwaves continue to pose a threat to coral reefs worldwide. However, refugia may help sustain coral populations. We used data from 30,000+ surveys worldwide, spanning from 2002 to 2020, to identify the environmental conditions that may characterize marine‐heatwave refugia. Refugia appear concentrated in the equatorial Coral Triangle, where marine heatwaves and coral bleaching were less severe than elsewhere. Reefs with high wave energy, current velocity, cloudiness, or turbidity also appear to be refugia. A global network of refugia, particularly those in the equatorial Coral Triangle, may sustain corals well beyond their forecasted demise by the end of the current century.

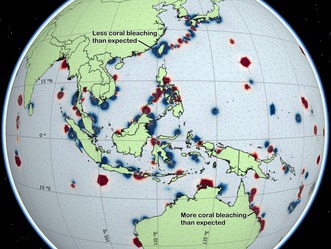

## 1 Introduction

Modern climate change has prompted one of the most pressing research questions in coral‐reef ecology: where will coral reefs persist through the Anthropocene? Indeed, modern climate change is increasing the frequency, intensity, and duration of marine heatwaves (Oliver et al. 2018; Frölicher et al. 2018; Laufkötter et al. 2020; Henley et al. 2024; Marcos et al. 2025), which are periods of anomalously high regional ocean temperatures (Oliver et al. 2021) that cause mass coral bleaching and mortality (Glynn 1993; Brown 1997; Hoegh‐Guldberg 1999; Hoegh‐Guldberg et al. 2007; Hughes et al. 2017). As marine heatwaves are predicted to continue occurring over the next century (Oliver et al. 2018; Frölicher et al. 2018; Laufkötter et al. 2020), numerous coral bleaching forecasts predict that equatorial coral reefs (i.e., those between ~12° north and south of the Equator; Wallace 1878) are the reefs most threatened by marine heatwaves (Teneva 2012; van Hooidonk et al. 2013, 2014, 2016; McManus et al. 2020; Mellin et al. 2024). However, such predictions are inconsistent with latitudinal patterns of marine heatwave characteristics (Oliver et al. 2018, 2021; Holbrook et al. 2019; Jacox et al. 2020; Sen Gupta et al. 2020), thermal tolerance (Sunday et al. 2011, 2019; Dixon et al. 2015; Pinsky et al. 2019), and the historical and modern responses of many marine organisms to acute thermal stress (Penn et al. 2018; van Woesik 2025; Sully et al. 2019). Here, we challenge the hypothesis that equatorial coral reefs are the reefs most threatened by marine heatwaves and identify the environmental conditions that may support equatorial refugia for corals.

Approximately 252 million years ago, ~7 million years before scleractinian corals evolved (Stanley 1988; Stanley and Fautin 2001), global warming led to the end‐Permian extinction (Payne and Clapham 2012; Sun, Farnsworth, et al. 2024), which was the most severe extinction event since life on Earth began ~3.8–4.3 billion years ago (Dodd et al. 2017). Although the end‐Permian event drove ~96% of marine life to extinction, geological records showed that ocean warming and extinction of many marine species were lowest near the Equator (Penn et al. 2018). Following the evolution of scleractinian corals ~245 million years ago, the enhanced survival of equatorial marine life during historical thermal stress repeated following the hyperthermal events of the end‐Triassic extinction, the Toarcian Oceanic Anoxic Event, and the Paleocene‐Eocene Thermal Maximum ~201, ~183, and ~56 million years ago, respectively (van Woesik 2025). The enhanced survival of equatorial coral reefs during historical thermal stress appears to be repeating in modern times, as the severity of coral bleaching during marine heatwaves from 1998 to 2017 was positively related to absolute latitude (Sully et al. 2019). Indeed, marine heatwaves in the western Pacific Ocean, where most coral reefs are located (Darwin 1842; Lyons et al. 2024), generally have the lowest intensities and shortest durations near the Equator (Oliver et al. 2018, 2021; Holbrook et al. 2019; Jacox et al. 2020; Sen Gupta et al. 2020). Moreover, the thermal tolerance of many marine, freshwater, and terrestrial species appears to be highest near the Equator (Sunday et al. 2011, 2019; Dixon et al. 2015; Pinsky et al. 2019).

Refugia are generally considered to be habitats that serve as biodiversity safe havens, where organisms can survive during periods of environmental stress, disturbance, or climatic change, and from which populations may later expand when surrounding conditions improve (Rull 2009; Stewart et al. 2010; Keppel et al. 2012). However, no refugium is necessarily permanent across space and time, and refugia vary in their capacity to buffer environmental change. Indeed, the spatial and temporal scale of a refugium depends on the magnitude of environmental change and the extent to which that change is buffered, ranging from fine to coarse spatial scales and from short‐ to long‐term durations relative to the mobility and generation time of the species it sustains (Levin 1992; Stewart et al. 2010; Keppel et al. 2012; Hannah et al. 2014). Refugia are, therefore, inherently subject to dynamic changes. Networks of such dynamic refugia have been considered for corals in the context of historical glacial and hyperthermal events (Stanley 1988; Potts and Jacobs 2000; Mosblech et al. 2011; van Woesik 2025) and in the face of modern marine heatwaves (van Woesik et al. 2012; Cacciapaglia and van Woesik 2016; Cinner et al. 2016; Sully and van Woesik 2020; Sully et al. 2022; Lachs, Humanes, et al. 2024; Sun, Steinberg, et al. 2024).

Environmental conditions that may locally moderate coral bleaching by reducing thermal stress during marine heatwaves include high cloud coverage (Mumby et al. 2001; Cheung et al. 2025), high water‐flow rates (Nakamura and van Woesik 2001; Nakamura et al. 2005; Cheung et al. 2025), high wave energy (Glynn 1993), turbidity (van Woesik et al. 2012; Cacciapaglia and van Woesik 2016; Teixeira et al. 2019; Sully and van Woesik 2020; Rosedy et al. 2023; Cheung et al. 2025), and high reef rugosity (Liesegang et al. 2025). As high water flow and shear stress toward the tops of reefs may increase mass transfer (Lenihan et al. 2008), which enhances survival during thermal stress (Nakamura and van Woesik 2001), areas of reefs that are shallower than the surrounding reef may have less coral bleaching than areas of reefs that are deeper than the surrounding reef. Reduced irradiance may also decrease the amount of thermal stress corals experience (Takahashi et al. 2004). Irradiance can be reduced by several factors, such as the angle of the reef slope relative to the sea surface (Brakel 1979), the degree to which the reef slope faces away from the Equator (Goreau and Goreau 1973), turbidity (van Woesik et al. 2012), and tidally driven currents that keep sediments in suspension (Kleypas 1996). Local moderation of coral bleaching may also arise from ecological memory—the capacity for past events to influence a phenotypic response to present stimuli (Peterson 2002). For example, historical marine heatwaves may moderate bleaching responses of corals to subsequent marine heatwaves (Thompson and van Woesik 2009; Sully et al. 2019) by selecting for thermally tolerant species (Loya et al. 2001) and thermally tolerant individuals (Howells et al. 2025).

We used a global database of coral bleaching (van Woesik and Kratochwill 2022), a comprehensive suite of variables, and Bayesian modeling (Rue et al. 2009; Lindgren and Rue 2015; Kubinec 2023) to reveal the environmental conditions that may characterize marine‐heatwave refugia for coral reefs worldwide. We also evaluated how ecological memory may influence the geographical responses of coral reefs to marine heatwaves. Together, our study challenges the hypothesis that equatorial coral reefs are the reefs most threatened by marine heatwaves and identifies the environmental conditions that may moderate coral bleaching based on a comprehensive global analysis of coral bleaching over the past two decades.

## 2 Materials and Methods

### 2.1 Data Collection

**FIGURE 1:**
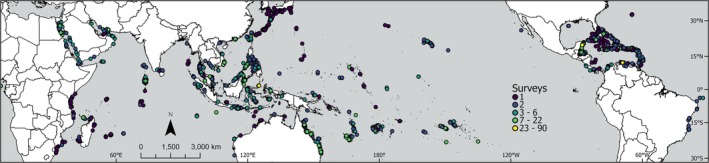
Locations of the 30,266 coral‐reef surveys conducted at 8728 sites (≤ 20 m depth) across 81 countries from 2002 to 2020. The sites are colored according to the number of surveys. Map lines delineate study areas and do not necessarily depict accepted national boundaries.

Coral bleaching was estimated during field surveys as the percentage of hard‐coral colonies that were bleached at study sites (van Woesik and Kratochwill 2022). As the taxonomic identity of corals and the relative abundance of each coral taxon were not recorded during surveys, the severity of coral bleaching at each site was estimated and assessed at the scale of the overall hard‐coral assemblage. In total, we used coral‐bleaching data from 30,266 surveys conducted at 8728 sites (≤ 20 m depth), and located between 35° north and south of the Equator across 81 countries, from July 7, 2002, to August 15, 2020 (Figure 1). Here, a survey is a unique combination of a study site, survey date, and depth. Detailed descriptions of the coral‐bleaching data are available in van Woesik and Kratochwill (2022).

We examined the relationship of local and regional environmental conditions, habitat characteristics, the pre‐disturbance reef state, and sampling bias with coral bleaching, including (i) sea‐surface temperature (°C), (ii) sea‐surface irradiance (E m^−2^ day^−1^), (iii) the interaction between sea‐surface temperature and sea‐surface irradiance (°C E m^−2^ day^−1^), (iv) cumulative marine‐heatwave intensity (°C), (v) the average annual frequency of marine heatwaves (# year^−1^), (vi) turbidity (as *K*_d_490; m^−1^), (vii) surface‐current velocity (m s^−1^), (viii) wave energy (J m^−2^), (ix) tidal range (m day^−1^), (x) climatological cloud frequency (%), (xi) reef depth (m), (xii) reef rugosity (m), (xiii) reef slope (°), (xiv) polar alignment of reef aspect (unitless), (xv) reef bathymetric‐position index (m), (xvi) absolute latitude of a reef, (xvii) overall hard‐coral cover during surveys (%), and (xviii) sampling effort (# samples ecoregion^−1^ month^−1^ year^−1^).

#### 2.1.1 Sea‐Surface Temperature

The average sea‐surface temperature (°C) for the 30 days preceding each survey, at 5‐km resolution, was computed from daily sea‐surface temperatures provided by the *CoralTemp* v3.1 dataset (Skirving et al. 2020). This process began by computing the grid‐cell indices for all of the 8728 sites, and then isolating 11,412 unique grid‐cell‐survey‐date combinations. For each of these combinations, the file paths to the daily sea‐surface‐temperature files were precomputed for the 30 days leading up to each grid cell's survey date(s), resulting in 684,720 matching files whose existence was confirmed—6657 of which were unique. A parallel session was initialized while allocating all cores except one; each unique precomputed file path was opened once, and the sea‐surface temperature values were extracted for all the grid cells that depended on that file at any point in their 30‐day period. The resulting extracted data, with file‐level sorting, were sorted by the grid‐cell‐survey‐date combinations, and the 30‐day average sea‐surface temperature was computed for each combination.

#### 2.1.2 Detecting Marine Heatwaves

A marine heatwave is at least five consecutive days of positive sea‐surface temperature anomalies exceeding the 90^th^ percentile climatological baseline for each day of the year (Hobday et al. 2016). Therefore, daily sea‐surface temperature data, at 5‐km resolution, were extracted from *CoralTemp* v3.1 from January 1, 1985, to December 31, 2020 (Skirving et al. 2020). The data were extracted for the 4365 unique 5‐km grid cells that contained at least one survey. All the 36‐year time series were passed to the *ts2clm* method of the *heatwaveR* package (Schlegel and Smit 2018), which implements the definition of a marine heatwave developed by Hobday et al. (2016), to calculate the climatological mean and 90^th^ percentile sea‐surface temperature for each day of the year, at each grid cell, over the 30‐year climatological period January 1, 1985, to December 31, 2014. These results were then passed to the *detect_event* method of the *heatwaveR* package to detect all marine heatwaves that occurred in each grid cell. All surveys were assigned to an active marine heatwave if the field survey occurred during, or at most 14 days after, any detected marine heatwave, at the respective grid cells.

#### 2.1.3 *heatwaveR* Metrics

The onset rate (°C day^−1^), decline rate (°C day^−1^), and duration (days) of the active marine heatwaves and their average, maximum, and cumulative intensity (°C) over their total duration were obtained from the *detect_event* metrics. Instead of using the total duration of each active heatwave, we only considered the duration of the heatwaves leading up to each survey by subtracting the event's start date from the survey date. Similarly, instead of using the total cumulative intensity of each active heatwave, we only considered the cumulative intensity that accumulated leading up to each survey by summing the daily temperature anomalies that occurred before and on the survey day (e.g., Figure S1). If the survey occurred after the heatwave's end date but within the 14‐day window, then the total duration and total cumulative intensity were used in the analysis.

The average annual frequency of historical heatwaves was calculated as the number of heatwaves that occurred 0.5–15.5 years before each survey, divided by 15 years. Heatwaves that occurred within half a year leading up to surveys were excluded to ensure that modern heatwaves were not counted with historical heatwaves, and heatwaves that occurred more than 15.5 years before surveys were excluded to ensure that the time window for the earliest surveys in 2002 did not extend earlier than the beginning of the temperature record in 1985. As many of the *heatwaveR* metrics were collinear, for the analysis, we only retained heatwave frequency and the cumulative intensity of marine heatwaves leading up to surveys. The cumulative intensity of active and recently active heatwaves was used to assess the relationship between active heat stress and coral bleaching, whereas heatwave frequency was used to assess the relationship between historical heat stress and coral bleaching.

#### 2.1.4 Irradiance and Turbidity

Daily photosynthetically available radiation (i.e., irradiance) data at the sea surface, at 4‐km resolution, from July 4, 2002, to August 15, 2020, were extracted from the Aqua Moderate Resolution Imaging Spectrometer (Aqua‐MODIS) (Frouin and Pinker 1995; Frouin et al. 2012) for all the unique 4‐km grid cells that contained at least one survey. The data for the 30 days preceding each survey were extracted and averaged. The same approach was used for the turbidity data, using 60 days instead of 30 days, which were also provided by Aqua‐MODIS at the same spatio‐temporal coverage and resolution as irradiance (Austin and Petzold 1981; Werdell and Bailey 2005). A larger time‐averaging window was used for turbidity compared with irradiance to account for more days with missing data.

#### 2.1.5 Surface‐Current Velocity

Current velocities were estimated from the Global Ocean Physics Reanalysis (GLORYS; Jean‐Michel et al. 2021), which is provided by the Copernicus Marine Service. GLORYS provides estimates of eastward (u) and northward (v) current velocities (m s^−1^) daily, at 9.2‐km resolution and at 50 depths ranging from 0.494 to 5728 m, from December 31, 1992, to August 25, 2025. The GLORYS ocean‐current data were downloaded approximately at the sea surface (0.494 m) using the *subset* method of the *copernicusmarine* Python application programming interface. The u and v surface‐current velocities were isolated for all survey sites for 30 days leading up to each survey. The current‐velocity rates (s) were then estimated for each of the 30 days as the magnitude of the u and v components, as:

(1) $$s=\sqrt{u^{2}+v^{2}}.$$

The current‐velocity rates for each of the 30 days preceding the surveys were then averaged.

#### 2.1.6 Wave Energy

The average wave energy at each site, 60 days leading up to, and during, the surveys, was estimated from the significant wave height data provided by Copernicus Marine Service's Global Ocean Waves Reanalysis (Law‐Chune et al. 2021). Significant wave height is the average height of the top‐third highest waves, measured in meters, estimated every 3 h at 22‐km resolution globally from December 31, 1979, to April 30, 2023 (Law‐Chune et al. 2021). The significant wave‐height data were downloaded from the Copernicus Marine Science server via the *get* method of the *copernicusmarine* Python application programming interface. The raster grid cell for each site coordinate was calculated, and all the unique grid‐cell‐survey‐date combinations were isolated. The data were extracted in parallel for each of these combinations over the 60 days leading up to and during each survey, following similar logic to the extraction of the sea‐surface temperature data. Wave‐energy density (E; J m^−2^) was then estimated as (Stokes 1847; Longuet‐Higgins 1952; Dean and Dalrymple 1991):

(2) $$E=\frac{1}{8}\mathrm{\rho g}H_{s}^{2},$$

where ρ is the density of seawater (1025 kg m^−3^; Marcet 1819; Murray and Buchanan 1884), g is the gravitational acceleration (9.81 m s^−2^; Huygens 1673), and $H_{s}$ is the average significant wave height (m) for the 60 days leading up to each survey.

#### 2.1.7 Tidal Range

Tidal‐range data (m day^−1^), which is the difference between high and low tide in meters, were extracted, at 3.7‐km resolution, from the Global Tidal Range dataset (Table S1). The Global Tidal Range dataset was derived from the Finite Element Solution 2022 tidal model (Centre National d'Etudes Spatiales 2024), which uses altimetry and tidal gauge measurements from 1992 to 2020, and was calculated as twice the climatological tidal amplitude:

(3) $$R=2\times \left(M_{2}+S_{2}+K_{1}+O_{1}\right),$$

where R is tidal range, $M_{2}$ is the principal lunar‐semidiurnal constituent, $S_{2}$ is the principal solar‐semidiurnal constituent, $K_{1}$ is the lunisolar‐diurnal constituent, and $O_{1}$ is the lunar‐diurnal constituent (Doodson 1921; Devlin et al. 2017). The Finite Element Solution 2022 tidal model follows the Finite Element Solution 2014 tidal model (Lyard et al. 2021).

#### 2.1.8 Cloud Frequency

The climatological cloud frequency for each month and site, expressed as the percentage of days in each month when clouds were present, was extracted from the dataset developed by Wilson and Jetz (2016), which is based on daily daytime surface‐reflectance data, at 1‐km resolution, from February 2000 to March 2014.

#### 2.1.9 Topography

We calculated a suite of local topographic features from derived bathymetry estimates by Li et al. (2021). The bathymetry data were estimated at a 10‐m resolution, based on the 2019–2024 median harmonized surface reflectance from the Sentinel‐2 multispectral satellite. The light‐attenuation algorithm developed by Li et al. (2021) can estimate depth down to 20 m; hence, we discarded surveys at sites deeper than 20 m. In their light‐attenuation algorithm, Li et al. (2021) assumed a fixed chlorophyll‐*a* concentration of 0.5 mg m^−3^ for all surface seawater. This assumption is reasonable to preserve the accuracy of the relative differences among the derived depths and, accordingly, of the estimated topography metrics within our relatively small kernel dimensions (900 and 2500 m^2^; see the subsequent paragraphs), as the concentration of chlorophyll‐*a* is relatively spatially homogeneous over hundreds of kilometers (Dasgupta et al. 2009). However, this assumption is not reasonable for accurately deriving absolute depths for all shallow‐water reefs, as the concentration of chlorophyll *a* varies considerably throughout the world's oceans (Feldman et al. 1989; Figure S2). Therefore, we retained the ground‐truthed depths from the Global Coral‐Bleaching Database (van Woesik and Kratochwill 2022) to assess the relationship between depth and coral bleaching.

The light‐attenuation algorithm to estimate bathymetry was translated from JavaScript to R using *rgee* (Aybar et al. 2020). A 70‐by‐70 m image patch of the derived bathymetry, centered on each site, was downloaded from Google Earth Engine using the *ee_as_rast* method of the *rgee* package. The rugosity, slope, aspect, northness, southness, and bathymetric‐position index (e.g., Figure S3) of each site were estimated from the derived bathymetry, also at a 10‐m resolution, using kernel‐convolution methods in the *MultiscaleDTM* R package (Ilich et al. 2023).

Reef slope, reef aspect, and reef northness were calculated using the *SlpAsp* method of *MultiscaleDTM* with a 30‐by‐30 m convolutional‐queen kernel. Reef slope is the inclination of a reef relative to the sea surface, where 0° is parallel to the sea surface and 90° is perpendicular to the sea surface. Reef aspect is the cardinal orientation that a reef faces downslope, where 0° is northward, 90° is eastward, 180° is southward, 270° is westward, and 359° is also northward. Northness is the north‐south component of the reef aspect and was calculated as the cosine of the aspect, ranging from −1 (south) to 1 (north). Southness is the inverse of northness and was calculated by multiplying the northness by −1, ranging from −1 (north) to 1 (south). We defined polar alignment as the southness of sites in the southern hemisphere and the northness of sites in the northern hemisphere, so that all positive values represent sites that face away from the Equator and all negative values represent sites that face toward the Equator.

Rugosity was calculated using the *AdjSD* method of *MultiscaleDTM* with a 3‐by‐3 cell (i.e., 30 by 30 m) convolutional‐square kernel. The *AdjSD* method fits an ordinary least‐squares plane to the nine cells in the focal window, computes the residual depth of each cell from the plane, and calculates the standard deviation of the residuals (Ilich et al. 2023). Therefore, rugosity is 0 m for flat reefs and increases as the reef become increasingly complex. Each site's bathymetric‐position index was calculated using the *BPI* method of *MultiscaleDTM* with a convolutional‐annulus kernel whose inner and outer radii were 20 m and 50 m, respectively. A positive bathymetric‐position index indicates reef habitats that are shallower than the surrounding reef (e.g., reef crests and patch reefs), a negative bathymetric‐position index indicates reef habitats that are deeper than the surrounding reef (e.g., back‐reef lagoons and reef channels), and a bathymetric‐position index equal to 0 indicates reef habitats that are the same depth as the surrounding reef (e.g., inner‐reef flats). *MultiscaleDTM* was designed for terrestrial data where elevation values decrease downslope. Therefore, the bathymetry data were multiplied by −1 to ensure that depth values also decreased downslope, preventing inversion of aspect, derivatives of aspect, and the bathymetric‐position index.

**FIGURE 2:**
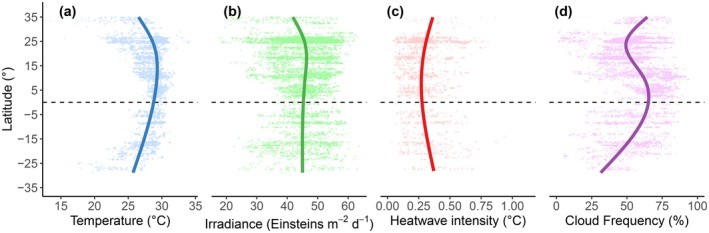
General latitudinal patterns of (a) sea‐surface temperature (°C), (b) sea‐surface irradiance (E m^−2^ day^−1^), (c) the average intensity of detected marine heatwaves over their full duration (°C), and (d) climatological cloud frequency (%), from 30,266 surveys conducted at 8728 sites (≤ 20 m), across 81 countries from 2002 to 2020. The raw data for each survey are the lightly colored dots. General latitudinal patterns are depicted by the colored curves, which are simple generalized additive models of the data estimated using restricted maximum likelihood and four knots (i.e., specific points along the range of latitude where the smooth function changes form).

For computational efficiency, the bathymetry‐raster convolutions were only performed for a small 49‐pixel (i.e., 70 by 70 m) image patch centered on each site coordinate. Before the convolutions, each image patch of the bathymetry and its corresponding site coordinate were projected into the appropriate Universal Transverse Mercator (UTM) zone using a custom UTM‐solver function. For each site, values for each topographical metric were obtained by passing the projected convolved‐image patches and the projected site coordinate to the *extract* method of the *terra* R package (Hijmans 2025). All topographic metrics were assumed to remain fixed over the study period. Depth values were converted back to the positive range after the topography data were extracted. Links to all the datasets we used are available in Table S1. All the data‐extraction code and extracted datasets are available at https://doi.org/10.6084/m9.figshare.30422674. An exploratory data analysis showed that several key variables had general latitudinal patterns (Figure 2).

### 2.2 Data Analysis

Data expressed as proportions are often modeled using a beta distribution (Pearson 1894) because the beta distribution is defined on the interval from zero to one, excluding zero and one. However, 15,685 of the 30,266 surveys recorded no coral bleaching (i.e., 0), which is undefined by the beta distribution. Therefore, we implemented a generalized linear ordered‐beta regression (Kubinec 2023), which is an extension of beta regression designed for zero‐and/or‐one‐inflated proportional data that (i) does not require transforming the data to the open interval (0, 1) (e.g., Smithson and Verkuilen 2006), (ii) does not require discrete (0 and 1) and non‐discrete (between 0 and 1) component models (e.g., hurdle models; Zuur et al. 2009), and therefore assumes that the discrete and non‐discrete values are generated by the same underlying process (in this case, marine heatwaves), and (iii) accounts for the continuous difference between the discrete and non‐discrete values (e.g., 0 is closer to 0.01 than to 0.5), which all considerably improve statistical inference (Kubinec 2023).

We incorporated ordered‐beta regression into *R‐INLA* (Lindgren and Rue 2015), which is a R package that estimates Bayesian posteriors (Bayes 1763) using integrated nested Laplace approximation (INLA; Rue et al. 2009). *R‐INLA* can explicitly model geographical space to account for spatial dependence in the response variable arising from spatial autocorrelation, and can reveal latent spatial patterns of the response variable not detected by the observed covariates (Lindgren et al. 2011; Bakka et al. 2018). Additionally, *R‐INLA* has a suite of random walk models to account for the temporal dependence of the response variable at multiple scales (Fahrmeir and Tutz 2001; Rue and Held 2005).

Here, explicit three‐dimensional modeling of geographical space in *R‐INLA* has three notable advantages. First, spatial autocorrelation of coral bleaching among sites can be accounted for by estimating the Matérn covariance of bleaching, which decays with increasing geodesic distance from other sites (Lindgren et al. 2011). Second, the nominal range can be estimated, which is the geodesic distance above which the Matérn covariance becomes negligible (Lindgren et al. 2011). In other words, the nominal range is the geodesic distance below which site‐scale coral bleaching is spatially autocorrelated among sites. Third, a spatial‐latent random effect can be estimated over the Earth and then interpolated at each site to account for the spatial autocorrelation of coral bleaching and all the residual geographical variation of coral bleaching that is unexplained by the rest of the model (Lindgren et al. 2011). These latent effects reveal spatially structured patterns of coral bleaching potentially attributable to unobserved environmental or physiological conditions (Lindgren et al. 2011). Projecting the spatial‐latent effect onto a map allows one to hypothesize which unobserved environmental or biotic variables may be influencing coral bleaching geographically. A positive spatial‐latent effect indicates where the observed coral bleaching was greater than expected by the model after accounting for the fixed effects, temporal effects, and the independent and identically distributed random effects, whereas a negative spatial‐latent effect indicates where the observed coral bleaching was less than expected by the model after accounting for the fixed effects, temporal effects, and the independent and identically distributed random effects.

*R‐INLA* probabilistically estimates all these parameters using stochastic partial differential equations over a Gaussian Markov random field (Rue and Tjelmeland 2002; Rue and Held 2005; Lindgren et al. 2011; Lindgren and Rue 2015), which was discretized as a three‐dimensional Delaunay triangulated mesh of all the site coordinates (Delaunay 1934) to avoid latitudinal distance distortions characteristic of planar geographic projections (Video S1). The resolution of the global mesh depends on the density of the sites. Areas with dense sampling were triangulated down to a 5‐km resolution to capture local‐scale autocorrelation of coral bleaching, whereas areas with sparse or no sampling (e.g., in open oceans) were triangulated up to a 333‐km resolution to reduce computational load. *R‐INLA* models the Earth as a unit sphere. Therefore, all of the site's two‐dimensional latitude‐longitude coordinates were converted to three‐dimensional Cartesian coordinates, and all the mesh parameters were converted to radians before being passed to the *R‐INLA* model (Lindgren et al. 2011). After the model was fitted, the Gaussian Markov random field and the nominal range were multiplied by Earth's volumetric mean radius of 6371 km (Dziewonski and Anderson 1981) to scale them back to geographic coordinates.

The zero‐inflated bleaching proportions (p) at study sites (s) were treated as spatio‐temporally dependent realizations of an ordered‐beta distribution. The coral bleaching data were treated as being spatio‐temporally dependent because samples collected close in space and time are inherently autocorrelated, which violates the independence requirement of a sample replicate (Hurlbert 1984; Legendre and Fortin 1989; Legendre 1993; Lennon 2000; Dormann 2007). Accordingly, the bleaching proportions were fitted to a Bayesian generalized linear mixed‐effects model with stationary spatial dependence and non‐stationary multi‐scale temporal dependence, defined on Earth's spherical domain and implemented in *R‐INLA*, as follows:

$$p_{i}\sim \text{oBeta}\left(\alpha ,\delta ,\gamma ,\mu ,\phi \right)$$

$$g\left(\mu -\varsigma \right)=\eta_{i}$$

(4) $$\begin{array}{c} \eta_{i}=\beta_{0}+\sum_{j=1}^{18}\beta_{j}z_{\mathrm{ij}}+u_{s}+\nu_{y}+\nu_{\mathrm{mh}}+o_{k}+l_{s}+d_{n}+\varepsilon_{i}, \end{array}$$

where $p_{i}$ is observation i of coral‐bleaching severity, expressed as a proportion (where i = 1–30,266); oBeta is the ordered‐beta distribution; α is the probability of observing no bleaching, defined as 1 minus the linear predictor given ς = $k_{1}$; δ is the probability of observing some bleaching, defined as the linear predictor given ς = $k_{1}$ minus the linear predictor given ς = $k_{2}$, which is then multiplied by the probability of the beta distribution given the linear predictor and ς = 0; γ is the probability of observing complete bleaching, defined as the linear predictor given ς = $k_{2}$; μ and ϕ are the mean and precision of the beta distribution, respectively; g is the logit‐link function; ς is cutpoint 1 ($k_{1}$), cutpoint 2 ($k_{1}$; where $k_{1}<k_{2}$), or 0 conditional on the observed level of bleaching, where $k_{1}$ defines the proportion of bleaching below which observing no bleaching becomes more probable than observing some bleaching, whereas $k_{2}$ defines the proportion of bleaching above which observing complete bleaching becomes more probable than observing some bleaching (Figure S4); $\eta_{i}$ is the linear predictor for observation i of coral bleaching; $\beta_{0}$ is the intercept; $\beta_{j}$ is the distribution of coefficient j for the standardized fixed‐effect covariate or interaction j ($z_{\mathrm{ij}}$; where j = 1–18); $u_{s}$ is the stationary, spatially‐correlated latent‐random effect for all observations at site s (where s = 1–8728), considered as a stochastic‐partial‐differential‐equation model to account for pseudoreplication arising from spatial autocorrelation of coral bleaching and for residual geographical variation of coral bleaching unexplained by the rest of the model, modeled three‐dimensionally at a 5 km resolution; $\nu_{y}$ is the temporally‐autocorrelated random effect for all observations in year y (where y = 2002–2020), considered as a first‐order random walk to account for pseudoreplication arising from annual autocorrelation of coral bleaching; $\nu_{\mathrm{mh}}$ is the temporally‐autocorrelated random effect for all surveys in month m (where m = 1–12) and hemisphere h (where h = 1–2; Northern and Southern Hemisphere), considered as a cyclic first‐order random walk to account for pseudoreplication arising from seasonal autocorrelation of coral bleaching; $o_{k}$ is the independent and identically distributed random intercept (Laird and Ware 1982) for all surveys in ocean k (where k = 1–3) to account for variation in bleaching between oceans; $l_{s}$ is the independent and identically distributed random intercept for all surveys at site s to account for variation in bleaching between survey coordinates; $d_{n}$ is the independent and identically distributed random intercept for all surveys from data source n (where n = 1–9) to implicitly account for variance in estimated bleaching values from different survey methods and surveyors; and $\varepsilon_{i}$ is the measurement error of observation i of coral bleaching defined by a Gaussian white‐noise process $\left[\sim N\left(0,\sigma_{\varepsilon }^{2}\right)\right]$.

All covariates were screened for collinearity, and those that were not collinear (i.e., had Pearson correlation coefficient magnitudes less than 0.6; Pearson 1895) were retained (Figure S5) and standardized by subtracting their mean and dividing by their standard deviation. The standardized variables were fitted to the model to ensure the fixed‐effect coefficients would be on a comparable scale. The variable for the temperature‐irradiance interaction was constructed by standardizing temperature and irradiance separately and then multiplying them (Aiken et al. 1991). Coral cover at the time of sampling was obtained from the Global Coral‐Bleaching Database (van Woesik and Kratochwill 2022). Sampling effort was calculated as the number of samples collected in each ecoregion during each month of each year.

We used *R‐INLA*'s Bayesian framework to estimate a posterior credibility distribution for each covariate's fixed effect, while accounting for spatial autocorrelation, temporal autocorrelation, and all the unobserved environmental and physiological conditions that may have influenced coral bleaching geographically. Such a framework allowed us to infer linear relationships between coral bleaching and our environmental covariates. Variables whose 90% credibility intervals were negative, included zero, or were positive had negative, no, or positive relationships with bleaching, respectively. This framework also allowed us to test the interaction between temperature and irradiance (i.e., their combined relationship with bleaching), where if the interaction's 90% credibility interval was negative, included zero, or was positive, then the interaction had an antagonistic, additive, or synergistic relationship with bleaching, respectively.

Oliver et al. (2018, 2021), Jacox et al. (2020), and Sen Gupta et al. (2020) found that the intensity of marine heatwaves for all surface seawater between 35° north and south of the Equator is generally lowest near the Equator, except in the eastern Pacific Ocean. As spatial patterns in ecological systems often display characteristic variability according to the scale of investigation (Levin 1992), another *R‐INLA* model was built to test whether the intensity of marine heatwaves that specifically occurred on shallow coral reefs is also generally lowest near the Equator. Of all the 30,266 surveys at 8728 sites, 6803 surveys occurred during, or shortly after (i.e., within 14 days), a marine heatwave at 2965 sites, as detected by the *heatwaveR* package. Heatwave intensities are continuous, positive, and theoretically unbounded, though practically constrained by an upper limit that is increasing over time (Oliver et al. 2018), and were therefore fitted to a gamma distribution as follows:

$$I_{i}\sim \text{gamma}\left(k,\theta \right)$$

$$\theta =\frac{\mu }{k}$$

$$g\left(\mu \right)=\eta_{i}$$

(5) $$\eta_{i}=\beta_{0}+\beta_{L}z_{\mathrm{iL}}+u_{s}+\nu_{y}+\nu_{\mathrm{mh}}+l_{s}+\varepsilon_{i},$$

where $I_{i}$ is the average intensity over the full duration of detected heatwave i (where i = 1–6803); k, θ, and μ are the shape, scale, and mean of the gamma distribution, respectively; g is the log‐link function; $\eta_{i}$ is the linear predictor for detected heatwave i; $\beta_{0}$ is the intercept; $\beta_{L}$ is the distribution of the fixed effect for absolute latitude; $z_{\mathrm{iL}}$ is the standardized absolute latitude for observation i; $u_{s}$ is the stationary, spatially‐correlated latent‐random effect for site s (where s = 1–2965), considered as a stochastic‐partial‐differential‐equation model to account for pseudoreplication arising from spatial autocorrelation of heatwave intensity and for residual geographical variation of heatwave intensity unexplained by the rest of the model, modeled three‐dimensionally at a 5‐km resolution; $\nu_{y}$ is the temporally‐autocorrelated random effect for all observations in year y (where y = 2002–2020), considered as a first‐order random walk to account for pseudoreplication arising from annual autocorrelation of heatwave intensity; $\nu_{\mathrm{mh}}$ is the temporally‐autocorrelated random effect for all observations in month m (where m = 1–12) and hemisphere h (where h = 1–2; Northern and Southern Hemisphere), considered as a cyclic first‐order random walk to account for pseudoreplication arising from seasonal autocorrelation of heatwave intensity; $l_{s}$ is the independent and identically distributed random intercept for all surveys at site s to account for variation in heatwave intensity between survey coordinates; and $\varepsilon_{i}$ is the measurement error of observation i of a heatwave defined by a Gaussian white‐noise process $\left[\sim N\left(0,\sigma_{\varepsilon }^{2}\right)\right]$.

The global *R‐INLA* models, as presented in Equations (4) and (5) for coral bleaching and marine heatwaves, respectively, were processed using an Intel Xeon E5‐2660 v3 central processing unit at 2.60 GHz and with 64 GB of random‐access memory. Both *R‐INLA* models are provided as RDS files, which can be read into R using *readRDS* (see “Data Availability Statement”). Neither model considers geographic barriers in the marine environment (i.e., land). Therefore, the results over land in all the maps shown for the coral‐bleaching model were masked by superimposing the land over the spatial‐latent field. The models were built in R v4.4.2 and *R‐INLA* v25.1.23. Our data analysis code is available at https://doi.org/10.6084/m9.figshare.30422674.

## 3 Results

**FIGURE 3:**
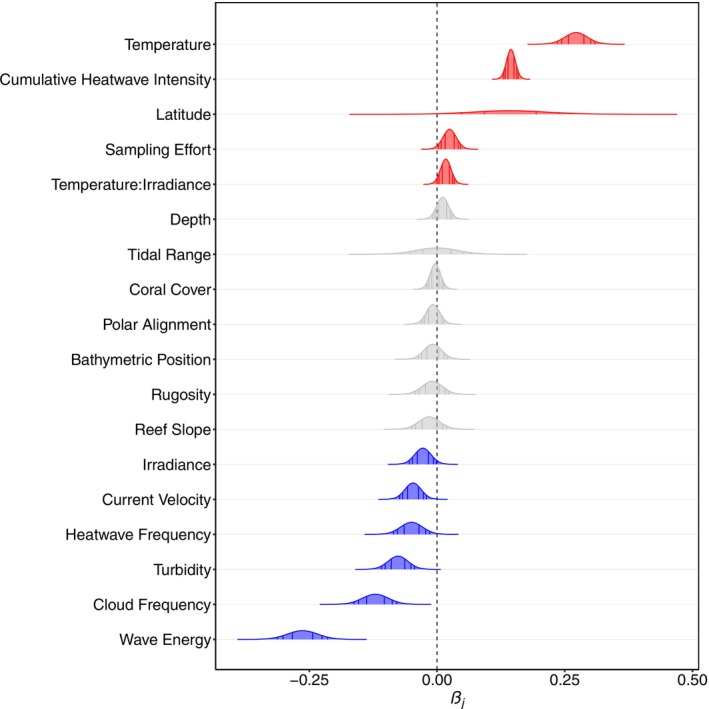
Posterior marginal distributions of the linear fixed‐effect coefficients from the coral‐bleaching model. Coral‐bleaching and environmental data from 30,266 coral‐reef surveys, at 8728 sites (≤ 20 m) across 81 countries from 2002 to 2020, were fitted to a Bayesian spatio‐temporal model, as in Equation (4). Variables with red coefficients exhibited positive relationships with coral bleaching. Variables with grey coefficients exhibited no relationships with coral bleaching. Variables with blue coefficients exhibited negative relationships with coral bleaching. The interaction with a red coefficient had a synergistic relationship with coral bleaching. All relationships are at least 90% probable. The vertical lines inside each distribution indicate the 50%, 80%, and 90% credibility intervals. Temperature is the 30‐day average before surveys at 5‐km resolution (°C); cumulative heatwave intensity is the sum of daily temperature anomalies before surveys that exceeded their day's 90^th^ percentile climatological baseline during an active marine heatwave lasting for at least five consecutive days at 5‐km resolution (°C); latitude is the reef site's absolute latitude (°); sampling effort is the number of samples collected in each ecoregion during each month of each year; the interaction between temperature and irradiance is the 30‐day average before surveys at 5‐ and 4‐km resolution for temperature and irradiance, respectively (°C E m^−2^ day^−1^); depth is the reef site's depth (m); tidal range is the difference between high and low tide at 3.7‐km resolution (m day^−1^); coral cover is the estimated percentage of the benthos that was covered by hard corals during surveys; polar alignment is the degree to which the reef's slope is oriented away from the Equator at 10‐m resolution (unitless); bathymetric position is the reef's depth anomaly relative to the surrounding bathymetry at 10‐m resolution (m); rugosity is the standard deviation of nine residual depths, each at 10‐m resolution, from the ordinary least‐squares 30‐m plane centered on the site at 10‐m resolution (m); reef slope is the reef's inclination relative to sea level at 10‐m resolution (°); irradiance is the 30‐day average before surveys at 4‐km resolution (E m^−2^ d^−1^); current velocity is the 30‐day average before surveys at 9.2‐km resolution (m s^−1^); heatwave frequency is the average number of heatwaves that occurred per year 0.5–15.5 years before surveys at 5‐km resolution; turbidity is the 60‐day average before surveys of the diffuse attenuation coefficient of light at 490 nm (i.e., *K*_d_490) at 4‐km resolution (m^−1^); cloud frequency is the climatological percentage of days in each month when clouds were present at 1‐km resolution; and wave energy is the 60‐day average before surveys at 22‐km resolution (J m^−2^). The observed and fitted values are plotted against each other in Figure S6.

**FIGURE 4:**
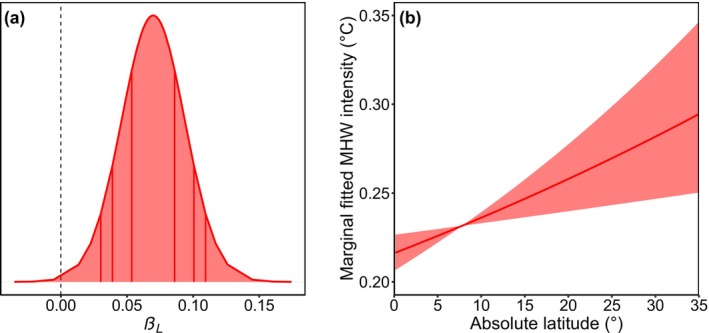
(a) Posterior marginal distribution of the linear fixed‐effect coefficient for absolute latitude and (b) the marginal fitted average marine‐heatwave (MHW) intensity based only on the fixed effect of absolute latitude (i.e., *β*_*L*_) and the fixed effect of the intercept (i.e., *β*_0_) from the marine‐heatwave model. The average intensity across the full duration of 6803 active marine heatwaves (and those that were recently active within 14 days of surveys) that were detected at 2965 sites (≤ 20 m), across 67 countries from 2002 to 2020, during 30,266 coral‐reef surveys at 8728 sites (≤ 20 m), across 81 countries from 2002 to 2020, was fitted to a Bayesian spatio‐temporal model, as in Equation (5). The 99% credibility interval for the fixed effect of absolute latitude is positive, indicating a strong positive relationship with the average intensity over the full duration of marine heatwaves that occurred on shallow coral reefs (a), which is illustrated with 95% credibility intervals (b). The vertical lines inside the posterior distribution indicate the 50%, 80%, and 90% credibility intervals (a). The observed values are mapped in Figure S7. The observed and fitted values are plotted against each other in Figure S8.

Sea‐surface temperature, the cumulative intensity of marine heatwaves leading up to surveys, absolute latitude, and sampling effort had positive relationships with coral bleaching (Figure 3). The interaction between temperature and irradiance had a synergistic relationship with coral bleaching (Figure 3). Depth, tidal range, coral cover at the time of sampling, and all topographic variables had no relationships with coral bleaching (Figure 3). Irradiance, current velocity, heatwave frequency, turbidity, cloud frequency, and wave energy had negative relationships with coral bleaching (Figure 3). Absolute latitude had a positive relationship with the average intensity of marine heatwaves that occurred on shallow coral reefs (Figure 4).

**FIGURE 5:**
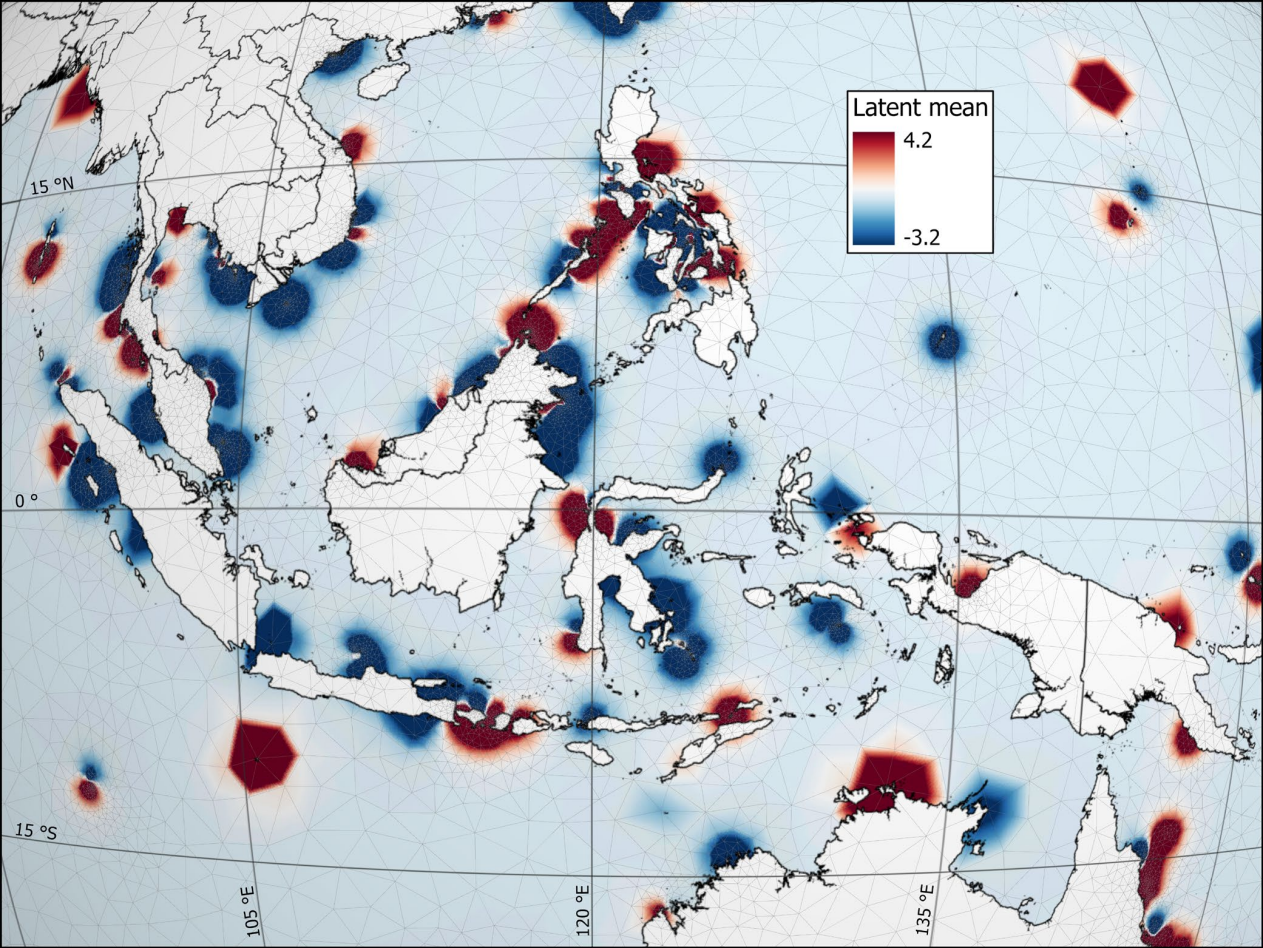
Spatial‐latent effects capture residual geographical variation of coral bleaching in the Coral Triangle from 2002 to 2020. Dark blue is where hard corals bleached less than expected by the model after accounting for the fixed effects, temporal effects, and the independent and identically distributed random effects, whereas dark red is where hard corals bleached more than expected by the model after accounting for the fixed effects, temporal effects, and the independent and identically distributed random effects. The spatial‐latent effect is expressed on the logit scale and was modeled at a 5‐km resolution over a three‐dimensional Gaussian Markov random field of Earth (i.e., the grey mesh). The scale bar is not included because the image is a perspective, top‐down view of Earth from space, and perspective distortion causes the map scale to vary throughout the image, as indicated by the curved longitude and latitude graticules. The distance between longitude graticules along the Equator is ~1665 km. North follows the longitude graticule upwards. Latent coral bleaching maps and their uncertainty are provided for all coral reef regions in Figures S10–S18. Map lines delineate study areas and do not necessarily depict accepted national boundaries.

Latent geographical patterns of coral bleaching were pervasive throughout all of the world's reefs (Figure S9). Notably, a negative spatial‐latent effect indicates where the observed bleaching was less than expected by the model after accounting for the fixed effects, temporal effects, and the independent and identically distributed random effects, whereas a positive spatial‐latent effect indicates where the observed bleaching was greater than expected by the model after accounting for the fixed effects, temporal effects, and the independent and identically distributed random effects. Negative spatial‐latent effects were pervasive in the Coral Triangle, off the coast of northwest Australia, the Pompey region of the Great Barrier Reef, mainland Japan, Taiwan, Vietnam, Myanmar, New Caledonia, Palau, Kosrae, Haiti, Bermuda, in the Seychelles, and in the northern Red Sea (Figure 5; Figures S9–S18). Positive spatial‐latent effects were pervasive on the Great Barrier Reef and in Hawaiʻi, the Maldives, and Brazil (Figures S9–S18). Negative and positive spatial‐latent effects were particularly heterogeneous throughout Fiji, French Polynesia, the Philippines, the Ryukyu Archipelago of Japan, the Florida reef tract, and the Caribbean (Figure 5; Figures S9–S18).

Site‐scale coral bleaching was, on average, spatially autocorrelated up to ~100 km (Figure S19). The log odds of coral bleaching were highest in 2006, 2010, 2014–2016, and in 2019 (Figure S20). The log odds of coral bleaching were also highest in October–December for the Northern Hemisphere and in March–June for the Southern Hemisphere (Figure S21).

## 4 Discussion

Sea‐surface temperature and the cumulative intensity of marine heatwaves had the strongest positive relationships with coral bleaching (Figure 3), supporting decades of well‐established theory, experiments, and global field observations showing that elevated temperatures are the primary cause of coral bleaching (Iglesias‐Prieto et al. 1992; Glynn 1996; Brown 1997; Hoegh‐Guldberg 1999; Loya et al. 2001; Berkelmans et al. 2004; Hughes et al. 2017; Sully et al. 2019; Shlesinger and van Woesik 2023). Irradiance, which has been experimentally shown to have a positive relationship with coral bleaching (Takahashi et al. 2004; Anthony et al. 2007), had a negative relationship with coral bleaching in our field study (Figure 3). Such an unexpected result may be because peak irradiance in the field typically precedes coral bleaching by several weeks to months because of thermal inertia (Liu et al. 1994; White et al. 1997) or because upregulation of photo‐acclimatory mechanisms during high irradiance moderated bleaching responses (Brown et al. 1994, 1999, 2000, 2002; Iglesias‐Prieto and Trench 1994; Gorbunov et al. 2001; Anthony and Hoegh‐Guldberg 2003; Hennige et al. 2008; Roth et al. 2010). Although the interaction between temperature and irradiance has been shown to be additive in bleaching experiments (Iglesias‐Prieto et al. 1992; Takahashi et al. 2004; Ban et al. 2014), the temperature‐irradiance interaction is also often assumed to have a synergistic relationship with coral bleaching (Coles and Jokiel 1978; Lesser et al. 1990; Drollet et al. 1994; Franklin et al. 2004; Lesser and Farrell 2004). Here, we show field evidence of such synergism (Figure 3). This result indicates that high temperatures combined with high irradiance exacerbate coral bleaching in the field, underscoring the importance of evaluating interactions between environmental conditions and biological processes (Dunne 2010; Côté et al. 2016). Our study also showed a negative relationship between coral bleaching and turbidity, which is consistent with the literature (van Woesik et al. 2012; Teixeira et al. 2019; Sully and van Woesik 2020; Cheung et al. 2025).

Our observation that marine heatwaves had the lowest average intensity on many equatorial reefs (Figure 4) is consistent with Oliver et al. (2018, 2021), Jacox et al. (2020), and Sen Gupta et al. (2020), who found that the intensity of marine heatwaves is generally lowest near the Equator, especially in the western Pacific Ocean. However, the warm water that pools near the Equator in the eastern Pacific Ocean during the El Niño phase of the El Niño‐Southern Oscillation is an exception to this global latitudinal pattern of heatwave intensity. The minimal intensity of marine heatwaves on most equatorial reefs, particularly those in the Coral Triangle, may be partially attributable to the Intertropical Convergence Zone that seasonally migrates between ~15° north and south of the Equator (Waliser and Gautier 1993; Schneider et al. 2014). While complex, the enhanced convective activity and evaporation in the Intertropical Convergence Zone facilitate the formation of clouds (Waliser and Gautier 1993; Schneider et al. 2014; Figure S22), which reduce short‐wave radiation and heating during marine heatwaves (Myers et al. 2018; Schmeisser et al. 2019; Rodrigues et al. 2019; Gao et al. 2020; Amaya et al. 2020; Zhao et al. 2021; Vogt et al. 2022; Dong et al. 2025), and may partially explain why coral bleaching has a positive relationship with latitude (Figure 3; Sully et al. 2019). Indeed, low‐latitude corals on Australia's Great Barrier Reef, with high cloud coverage, had minor (i.e., 0%–30%) bleaching even when exposed to major marine heatwaves in 2016, 2017, and 2020 (Cheung et al. 2025). However, many other factors may also regulate sea‐surface warming over reefs during marine heatwaves, including short‐term phases of various climate modes that vary geographically (Holbrook et al. 2019).

Our equatorial‐refugia hypothesis is, however, in stark contrast with coral bleaching forecasts predicting that marine heatwaves and coral bleaching will be most severe on equatorial reefs (Teneva 2012; van Hooidonk et al. 2013, 2014, 2016; McManus et al. 2020; Mellin et al. 2024). Coral bleaching forecasts use degree‐heating weeks and degree‐heating months to estimate the intensity of future marine heatwaves, which heuristically integrate simulated temperature anomalies over 12 weeks when they are 1°C above the hottest month's climatological mean temperature (Liu et al. 2003; Donner et al. 2005). Degree‐heating weeks and months, particularly months, are unreliable predictors of observed coral bleaching (McClanahan et al. 2019; Mason et al. 2025; Cheung et al. 2025), especially for large‐scale studies, likely because the extensive spatio‐temporal variability of coral thermal tolerance (McClanahan et al. 2020; Berkelmans and Willis 1999; Berkelmans 2002; García et al. 2024) is not accounted for by a spatio‐temporally fixed temperature anomaly threshold. By contrast, the heatwave definition developed by Hobday et al. (2016) dynamically integrates temperature anomalies above the 90^th^ percentile for each of the daily climatological thresholds, which, compared with degree‐heating metrics, better captures the spatio‐temporal variability of thermal extremes and the influence of Earth's seasonality on temperature anomalies. Accordingly, the degree‐heating‐week metric has been recently considerably refined by regionally optimizing the 1°C temperature anomaly threshold and the 12‐week temperature integration window (DeCarlo 2020; Whitaker and DeCarlo 2024). Furthermore, bleaching forecasts are also problematic because they attempt to predict ecological responses to marine heatwaves decades into the future, which is well beyond the predictable lead time for marine heatwaves of up to 1 year (Jacox et al. 2022). Our results emphasize the importance of using reliable metrics of environmental conditions to infer ecological patterns that align with field observations.

In addition to predicting that equatorial reefs are the reefs most threatened by marine heatwaves, coral bleaching forecasts also predict that without considerable emission reductions, acclimatization, or adaptation, most of the world's coral reefs will be severely degraded sometime during the current century (Donner et al. 2005; Donner 2009; Frieler et al. 2013; Logan et al. 2014, 2021; Ainsworth et al. 2016; Dixon et al. 2022; Lachs et al. 2023; Lachs, Bozec, et al. 2024; Bozec et al. 2025). However, such predictions are inherently the worst‐case scenario as they do not consider the moderating effects of the environment (Figure 3). Incorporating such moderating effects into coral bleaching forecasts (Cacciapaglia and van Woesik 2016; Storlazzi et al. 2020) may further reveal marine‐heatwave refugia across geographical scales and provide more accurate bleaching projections (Hannah et al. 2014; Lenoir et al. 2017; van Woesik et al. 2022; Klein et al. 2024; McClanahan 2024). Incorporating the principles of near‐term ecological forecasting into coral bleaching forecasts will improve our understanding of how the environment may regionally and locally moderate coral bleaching, worldwide, and allow us to accurately project the severity of coral bleaching during marine heatwaves (Clark et al. 2001; Dietze et al. 2018, 2024).

We report several environmental conditions that may locally moderate coral bleaching during marine heatwaves. For example, current velocity, turbidity, and wave energy had negative relationships with the severity of coral bleaching (Figure 3). The negative relationship between current velocity and coral bleaching is consistent with experiments (Nakamura and van Woesik 2001; Nakamura et al. 2005) and field observations (Cheung et al. 2025). Strong currents increase shear stress and mass transfer that reduce corals' thermal stress (Nakamura and van Woesik 2001) while also inducing water mixing that moderates regional temperatures during marine heatwaves (DiMassa et al. 2010; Green et al. 2019; Hsu et al. 2020; Storlazzi et al. 2020). Similar influences of shear stress and mass transfer are associated with high wave energy. The negative relationship between wave energy and the severity of coral bleaching is also consistent with several early anecdotes that bleaching was most severe during periods of calm seas, largely because of the buildup of the thermocline (Glynn 1993). High wave energy also disrupts the thermocline and causes cool‐water upwelling (Wunsch and Ferrari 2004). Moreover, low wind velocity lowers wind‐driven waves, water mixing, and cool‐water upwelling, increasing heating and the likelihood of coral bleaching.

Coral bleaching was also reduced on reefs that were historically exposed to frequent marine heatwaves (Figure 3), agreeing with many prior studies that have demonstrated increased resistance through successive marine heatwaves (Podestá and Glynn 2001; Thompson and van Woesik 2009; Guest et al. 2012; Sully et al. 2019; Shlesinger and van Woesik 2023; Lachs et al. 2023; González‐Barrios et al. 2023). Recently, Cheung et al. (2025) found that coral bleaching was reduced on reefs that were historically exposed to intense or recent heatwaves on the Great Barrier Reef. Such accumulation of coral reef resistance to marine heatwaves may arise from acclimatization (Gates and Edmunds 1999), phase shifts in community composition (Done 1992), and adaptation by natural selection (Darwin 1859; Côté and Darling 2010; Kenkel and Matz 2017; Howells et al. 2025), although these processes are not mutually exclusive.

Our study found no relationship between coral bleaching and any topographical variable. All reef topography metrics were estimated at 10‐m resolution, which is to date the highest available resolution of global bathymetry data. Such resolution of local‐reef geomorphology may still be too coarse to detect any relationships with coral bleaching, which would be optimally measured at the centimeter scale (Liesegang et al. 2025; Levin 1992). We also found no relationship between coral bleaching and depth, from 0 to 20 m. There was also no relationship between overall hard‐coral cover and the severity of coral bleaching, but such a relationship would likely change if the pre‐disturbance reef states were examined at a higher taxonomic resolution (Loya et al. 2001; Levin 1992). Tidal range also had no relationship with coral bleaching, perhaps because tidal range is typically homogeneous over hundreds of kilometers (Schwiderski 1980; Lyard et al. 2021), whereas site‐scale coral bleaching geographically varies among reef habitats (Berkelmans et al. 2004; van Woesik et al. 2012), all of which can be present over hundreds of kilometers (Kennedy et al. 2021; Levin 1992). Sampling effort in each ecoregion had a positive relationship with coral bleaching, likely because the detectability of bleaching increases with more surveys.

Although we examined many variables that are known to relate to the severity of coral bleaching, we may not have included all relevant variables in our analysis to capture all the geographical variation of coral bleaching. Moreover, as spatial patterns in ecological systems often display characteristic variability according to the scale of investigation, the variables we included may not have been estimated at the optimal spatial scales to capture all the geographical variation of coral bleaching (Levin 1992). Yet, one of the most powerful aspects of using *R‐INLA* is its ability to estimate spatial‐latent effects over the study domain to unveil residual geographical patterns of coral bleaching that were not accounted for by the observed fixed effects, temporal effects, and the independent and identically distributed random effects. Our results show that the majority of the Great Barrier Reef had positive spatial‐latent effects, where coral bleaching was higher than expected, suggesting that this region is geographically located where thermal stress is more impactful than expected (Cacciapaglia and van Woesik 2015; Wolanski et al. 2017). Positive spatial‐latent effects were also apparent in Hawai‘i, the northern Philippines, most of the Caribbean, the Maldives, and in Brazil. Negative spatial‐latent effects, where coral bleaching was lower than expected, were apparent in Taiwan and the southern Ryukyu Islands, mainland Japan, the Pompey region in the Great Barrier Reef, off the coast of northwest Australia, the Coral Triangle, Vietnam, Myanmar, New Caledonia, Palau, Kosrae, Haiti, Bermuda, in the Seychelles, and in the northern Red Sea. Explicitly modeling geographical space using *R‐INLA* also revealed that site‐scale coral bleaching on the world's shallow reefs was spatially autocorrelated up to ~100 km, which is very similar to correlation distances of ~100, ~140, and ~100 km for site‐scale coral bleaching on the Great Barrier Reef during marine heatwaves in 1998, 2002, and 2016, respectively (Dietzel et al. 2021).

The strong negative spatial‐latent effects in Taiwan and the southern Ryukyu Islands in Japan (Figure S13) may be related to hyper‐exposure to typhoons compared with other reefs in the world (Carrigan and Puotinen 2011). The reefs of the southern Ryukyu Islands have adjusted to high‐typhoon activity, showing minor colony breakage and no change to reef framework with typhoon overpass (R.v.W., personal observation). These structurally stable reefs may instead benefit from typhoons during marine heatwaves, as typhoons cool shallow water through upwelling (Manzello et al. 2007; Carrigan and Puotinen 2011, 2014). The northern Red Sea also had negative spatial‐latent effects, but the southern Red Sea had positive spatial‐latent effects (Figure S14). While we are hesitant to attribute this spatial pattern of latent coral bleaching in the Red Sea to differential resistance, our spatial‐latent results are consistent with Osman et al. (2018), who found that corals in the northern Red Sea are far more tolerant of heat stress than those in the southern Red Sea. We also found a strong negative spatial‐latent effect in the Pompey region of the Great Barrier Reef (Figure S12), which corresponds to a purported marine‐heatwave refugia (Sun, Steinberg, et al. 2024). Although current velocity was an observed variable in our study, the entire reef complex of the Pompey region is ~200 km long by ~90 km wide and persistently experiences upwelling of cool water that is flushed through the reefs by strong tidally driven currents (Sun, Steinberg, et al. 2024), which was unobserved by the much finer 9‐km resolution current velocity data in our study.

There may also be biological reasons as to why there were numerous reefs with negative spatial‐latent effects in the equatorial Coral Triangle (Figure 5). Indeed, many low‐latitude marine, freshwater, and terrestrial species have higher thermal tolerance compared with higher‐latitude species (Sunday et al. 2011, 2019; Dixon et al. 2015; Pinsky et al. 2019). The persistence of corals on equatorial reefs through historical and modern thermal‐stress events is likely in part because the most stable regional environments are near the Equator (Trewartha 1954). Stable equatorial environments have low selective pressure, leading to high species richness (Wallace 1878; Pianka 1966). Likewise, low‐latitude coral reefs have higher within‐population genetic diversity than reefs at higher latitudes (Miller and Ayre 2008; Underwood 2009; Noreen et al. 2009; Thomas et al. 2017). High among‐ and within‐species diversity at and near the Equator enhances the probability that some equatorial corals will have allelic variants that bestow thermal resistance. Indeed, marine ectotherms live closest to their thermal maximum near the Equator, where thermal tolerance is highest (Pinsky et al. 2019). Furthermore, low‐latitude ecosystems were refugia during past climate change (Hampe and Petit 2005).

Here, we formalize the coral‐bleaching equatorial‐refugia hypothesis and challenge the hypothesis that equatorial reefs will be the first casualties of marine heatwaves. Indeed, we found that the average intensity of marine heatwaves is lowest on most equatorial reefs, potentially because they are persistently shielded from extreme insolation by frequent cloud coverage in the Intertropical Convergence Zone. Therefore, except for the eastern Pacific Ocean, if marine heatwaves continue to be weakest on equatorial reefs, intense heatwaves at high latitudes may constrict corals toward the equatorial Coral Triangle. However, if the corals' thermal maxima are first exceeded near the Equator through long‐term warming of ambient sea‐surface temperatures (Pinsky et al. 2019), corals may migrate toward the poles in some regions (Precht and Aronson 2004; Greenstein and Pandolfi 2008; Yamano et al. 2011; Poloczanska et al. 2016; Kumagai et al. 2018), up to a light‐limiting latitude (Grigg 1982; Kleypas et al. 1999; Muir et al. 2015). Such poleward range shifts would counter any constrictions of corals toward the equatorial Coral Triangle following intense marine heatwaves. Moreover, the probability of occurrence of marine heatwaves is forecasted by some researchers to increase most rapidly near the Equator (Frölicher et al. 2018). If such a scenario manifests and equatorial marine heatwaves become intense, some heatwave refugia in the Coral Triangle could collapse. Yet, corals may persist during marine heatwaves in other heatwave refugia that experience high wave energy, high current velocity, high cloud frequency, or turbidity. Corals may also persist during marine heatwaves on reefs that previously endured many heatwaves. We may find that a dynamic, global network of refugia, especially those concentrated in the equatorial Coral Triangle, will help sustain coral metapopulations well beyond their thermodynamically forecasted demise from marine heatwaves by the end of the current century.

## Author Contributions

**Zachary Ferris:** conceptualization, data curation, formal analysis, funding acquisition, investigation, methodology, software, visualization, writing – original draft, writing – review and editing. **Andrew S. Walker:** conceptualization, software, writing – original draft, writing – review and editing. **Håvard Rue:** methodology, software, writing – review and editing. **Robert van Woesik:** conceptualization, funding acquisition, project administration, resources, software, supervision, writing – original draft, writing – review and editing.

## Conflicts of Interest

The authors declare no conflicts of interest.

## Supporting information


**Data S1:** gcb70594‐sup‐0001‐DataS1.pdf.

**Video S1:** gcb70594‐sup‐0002‐VideoS1.mp4.

## Data Availability

All code and derived data are available at https://doi.org/10.6084/m9.figshare.30422674 and at https://github.com/InstituteForGlobalEcology/Ferris‐et‐al‐2025‐GCB.
