# Supplementary material for: Coral Bleaching: The Equatorial‐Refugia Hypothesis
Source: Glob Chang Biol. 2025 Nov 17;31(11):e70594. doi: 10.1111/gcb.70594 (PMC12621085; doi:10.1111/gcb.70594)
Supplement: Supplementary file 1 — Data S1: gcb70594‐sup‐0001‐DataS1.pdf. [file GCB-31-e70594-s002.pdf]

Supplementary Information

Coral bleaching: the equatorial-refugia hypothesis

Z. Ferris, A. Walker, H. Rue, R. van Woesik

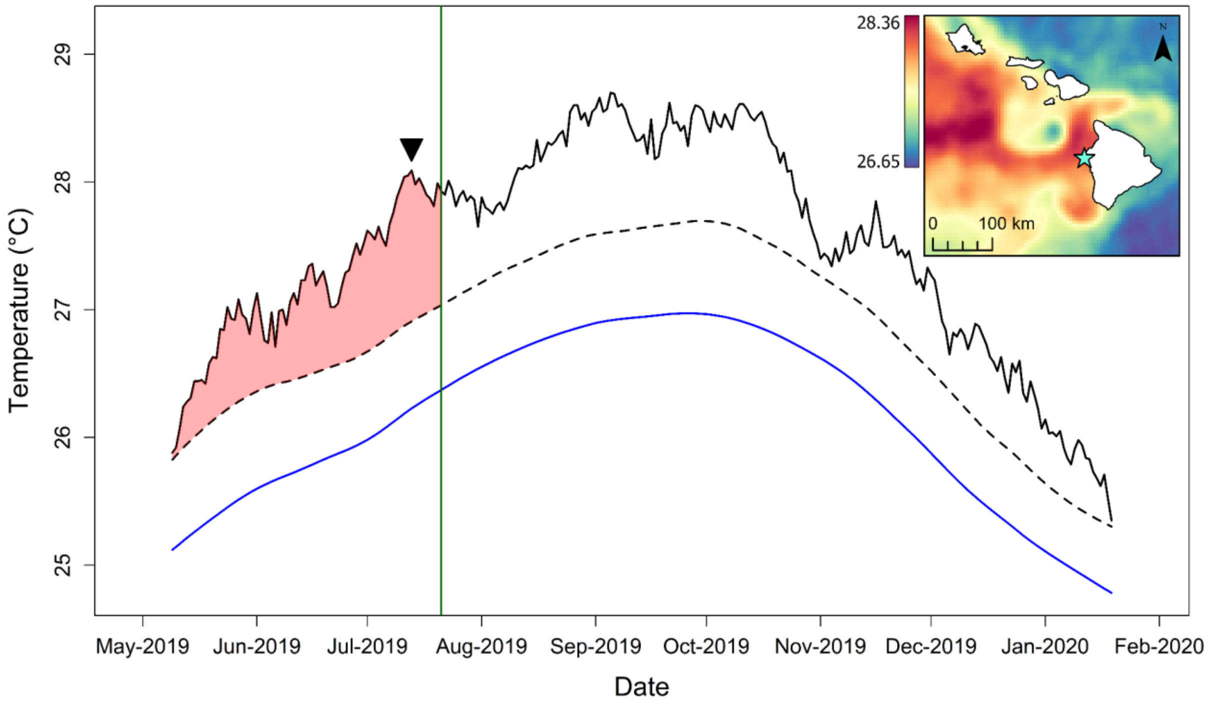

Supplementary Figure S1. Time series of daily temperature for the longest marine heatwave that was active during one of our surveys, demonstrating how we calculated the cumulative intensity of marine heatwaves leading up to surveys. A marine heatwave (i) began on May 9, 2019, at a study site in Hawaii (blue star) that was surveyed 74 days later on July 21, 2019 (vertical green line), (ii) peaked (relative to the 90% climatological threshold; black-dotted line) on July 13, 2019 (black triangle), and (iii) ended on January 19, 2020, lasting for a total of 256 days. The jagged black line is the temperature for each day over the full duration of the marine heatwave. The blue line is the climatological mean sea-surface temperature for each day from January 1, 1985, to December 31, 2014. The inset shows the sea-surface temperature on the peak date of the marine heatwave for most of the surrounding main Hawaiian Islands. The maximum temperature in the inset (i.e., 28.36 °C) is slightly higher than the temperature on the peak date of the heatwave, because the temperature time series is for the 5-by-5 km grid cell that encompasses the study site. The total area of the red shading represents the cumulative intensity of the marine heatwave that accrued, leading up to the survey. All the

active marine heatwaves in this study were detected using the *heatwaveR* package (Schlegel & Smit, 2018), which implements the definition of a marine heatwave developed by Hobday et al. (2016). Map lines delineate study areas and do not necessarily depict accepted national boundaries.

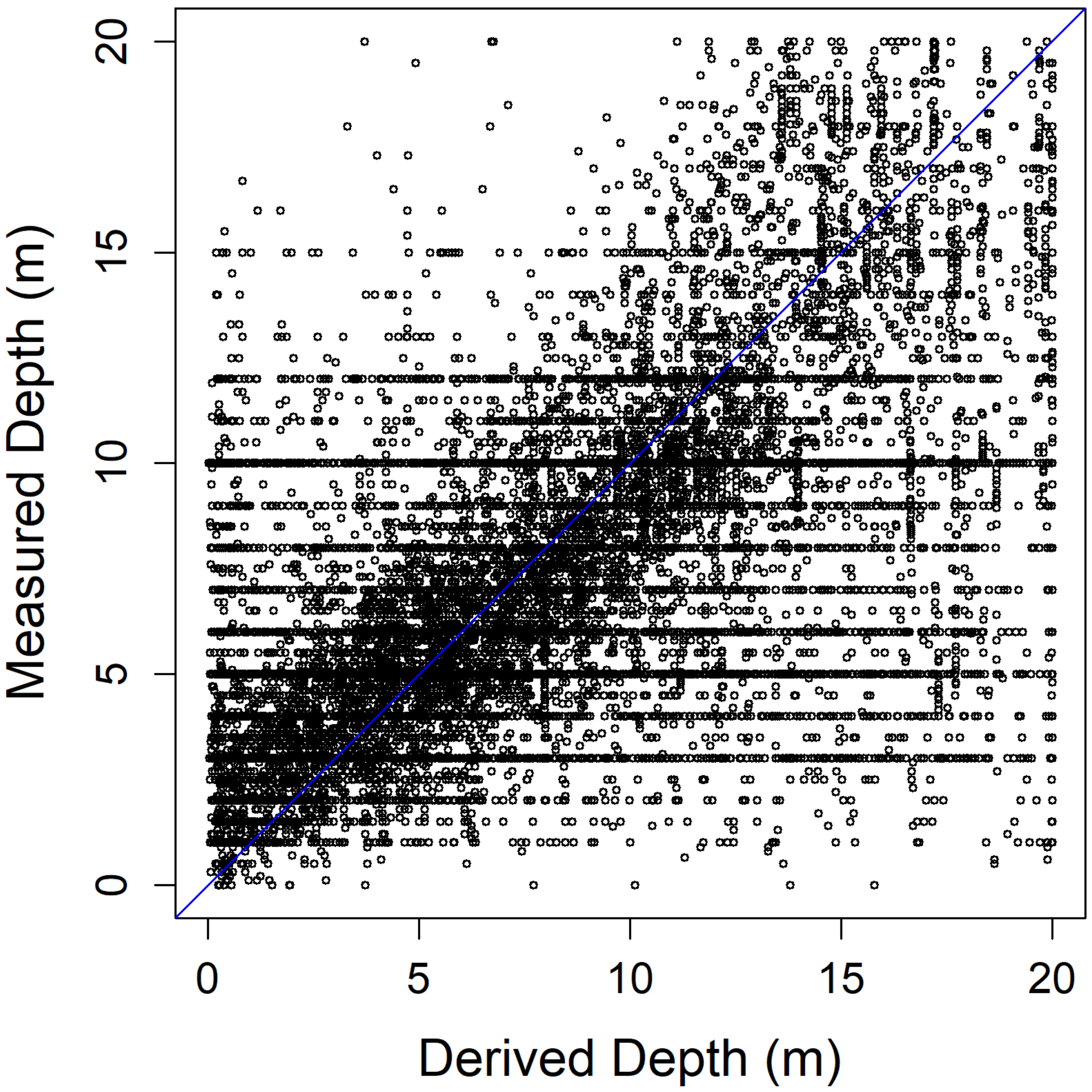

Supplementary Figure S2. The measured depth (m) against the derived depth (m) from 8,728 sites (≤20 m) across 81 countries. The derived depth using the algorithm developed by Li et al. (2021) is accurate for many reefs shallower than ~12 meters that had a chlorophyll-*a* concentration close to the assumed fixed value of 0.5 mg m^{-3}. However, the derived depth is inherently inaccurate for reefs that had a chlorophyll-*a* concentration that considerably deviated from 0.5 mg m^{-3}. The blue line indicates a one-to-one relationship.

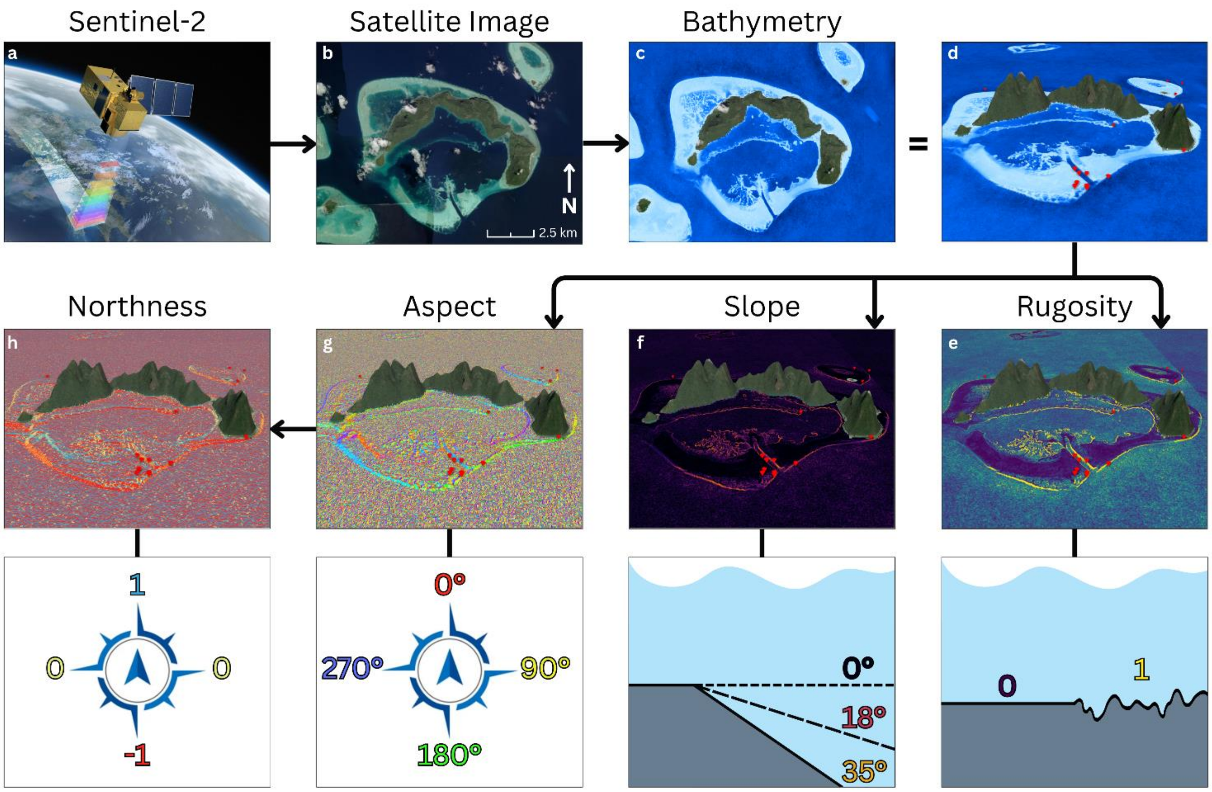

Supplementary Figure S3. Workflow for calculating topography from Sentinel-2-derived bathymetry at 8,728 sites (≤20 m) across 81 countries. (a) A rendering of the Sentinel-2 satellite obtained from the European Space Agency, **(b)** a cropped satellite image of an example reef at Bodgaya Island and Boheydulang Island (4.6 °N, 118.75 °E) in Borneo, Southeast Asia, **(c)** the derived bathymetry of **(b)** where light blue is zero meters and dark blue is twenty meters, **(d)** an oblique three-dimensional view of **(c)** where the red cones are the locations of 14 of our sites, and the **(e)** reef rugosity, **(f)** reef slope, **(g)** reef aspect, and **(h)** northness of **(d)**. Here, northness is also polar alignment because these islands are in the northern hemisphere. Map lines delineate study areas and do not necessarily depict accepted national boundaries.

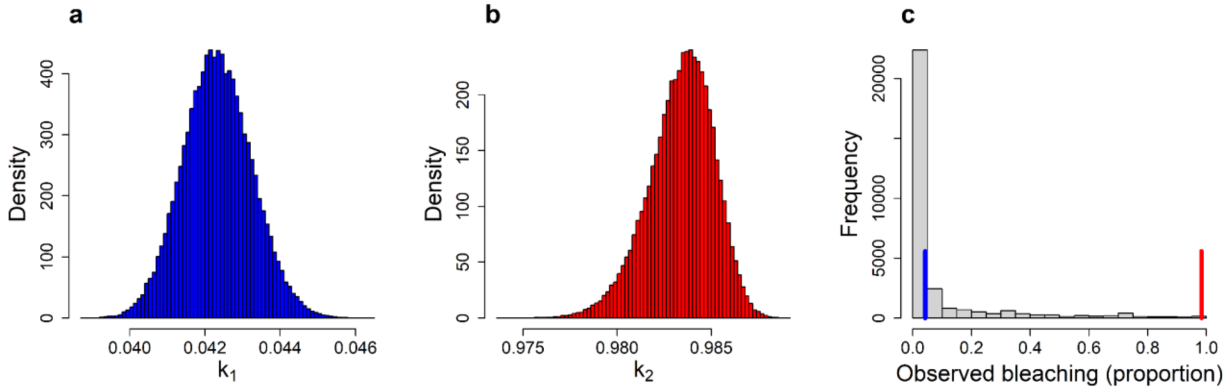

Supplementary Figure S4. Posterior distributions of the ordered-beta cutpoints from the *R-INLA* model. (a) The posterior distribution of cutpoint 1, **(b)** the posterior distribution of cutpoint 2, and **(c)** the distribution of the observed coral-bleaching data with the average of cutpoints 1 and 2 indicated by the vertical blue and red lines, respectively.

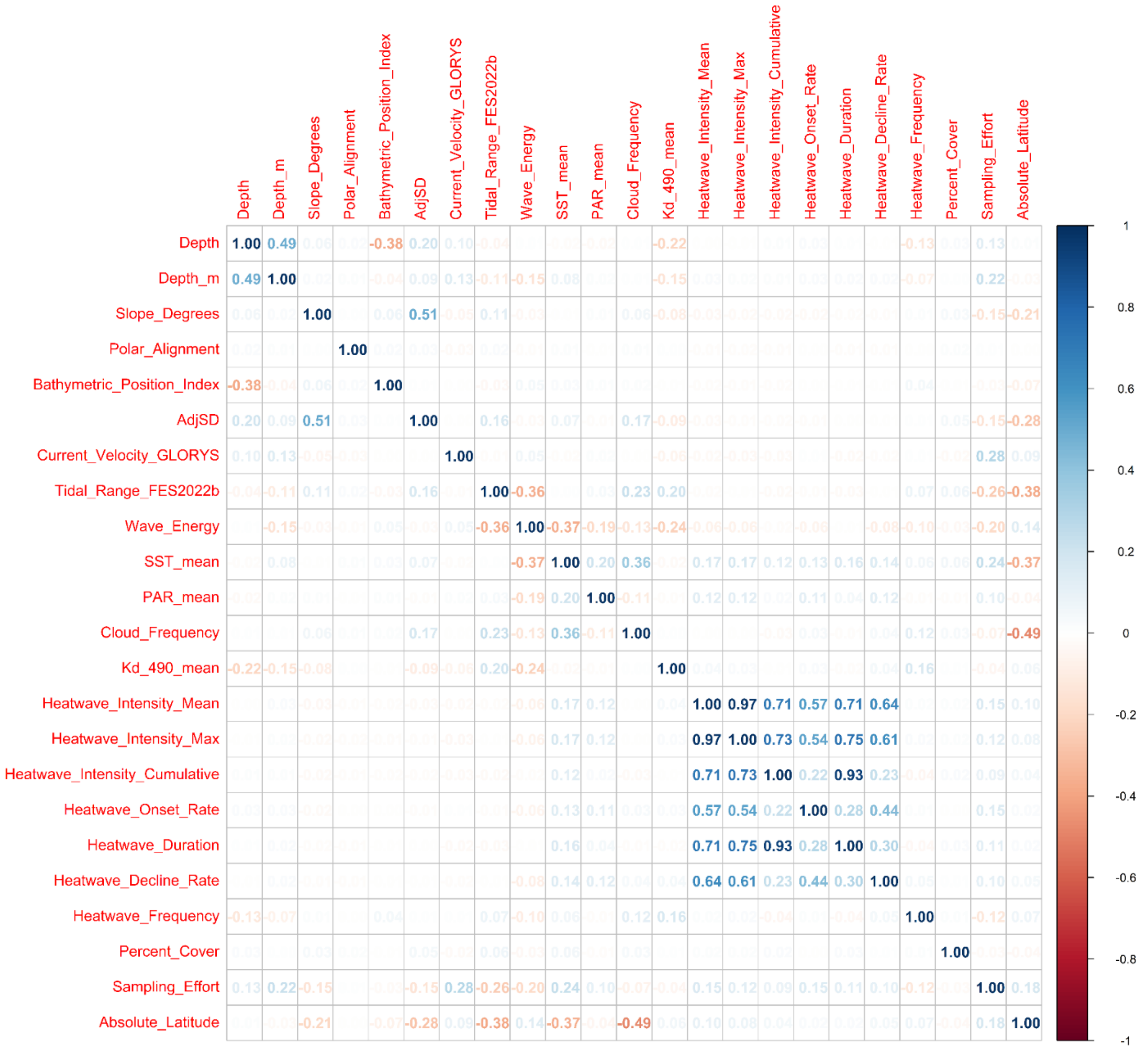

Supplementary Figure S5. Correlation coefficients between the candidate variables for the coral bleaching model from 30,266 coral-reef surveys at 8,728 sites (≤20 m) across 81 countries from 2002 to 2020.

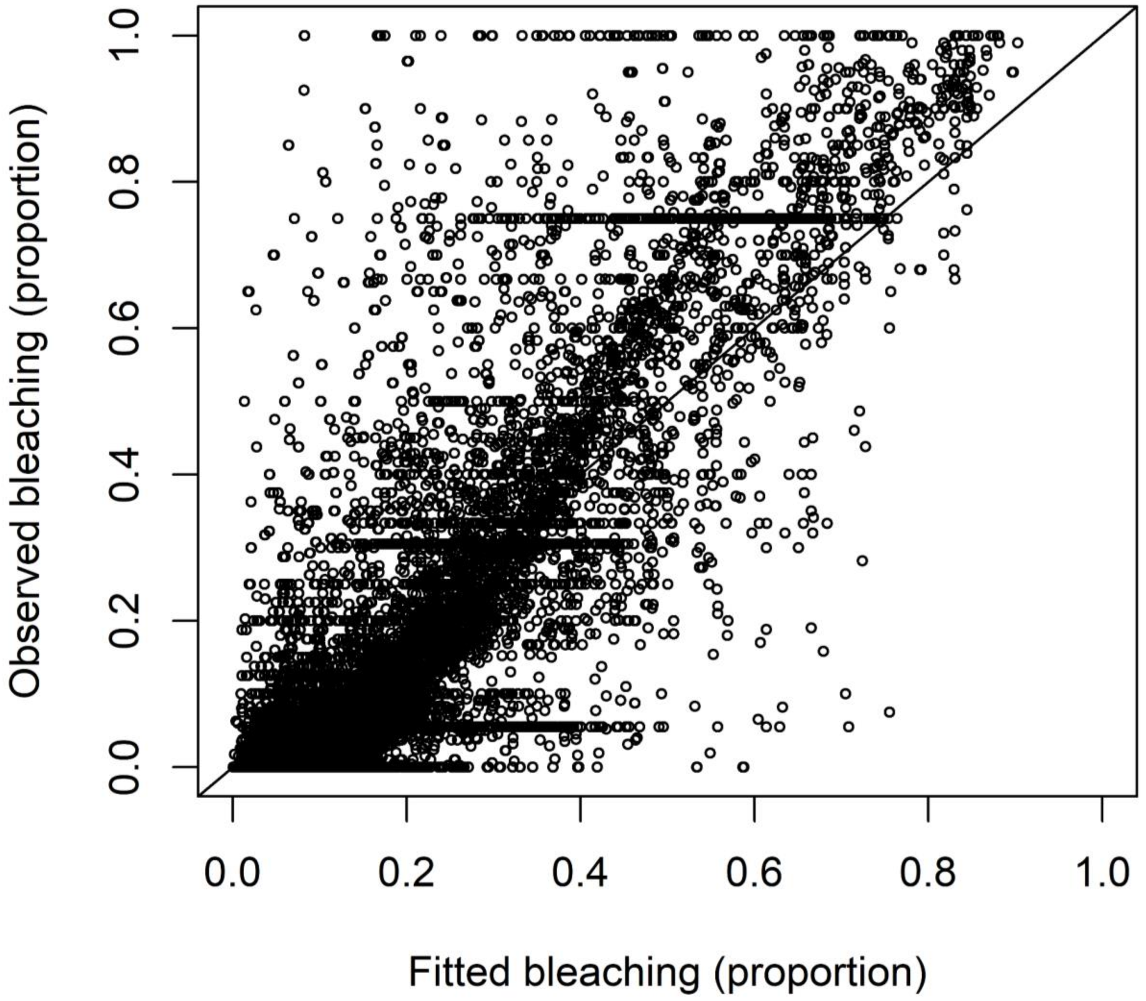

Supplementary Figure S6. The observed versus the fitted coral bleaching from Equation 4.

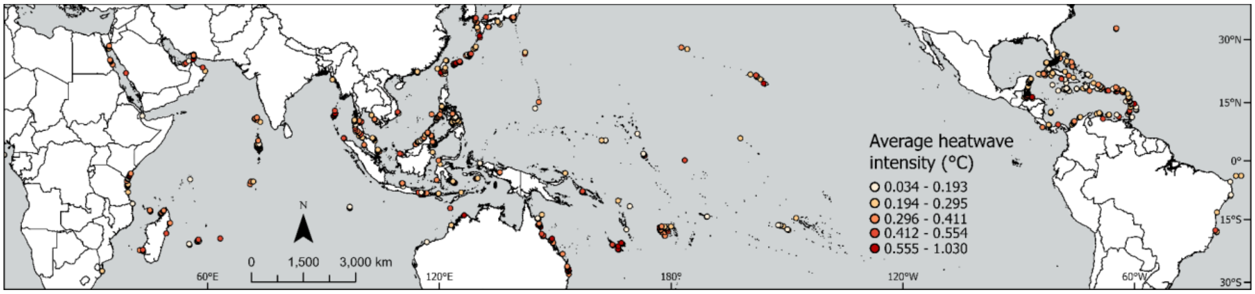

Supplementary Figure S7. The average intensity of 6,803 marine heatwaves detected at 2,965 sites in the Global Coral-Bleaching Database from 2002–2020. Sites are colored by the average heatwave intensity. The most intense heatwave is shown for sites that had numerous detected heatwaves during the study period. Map lines delineate study areas and do not necessarily depict accepted national boundaries.

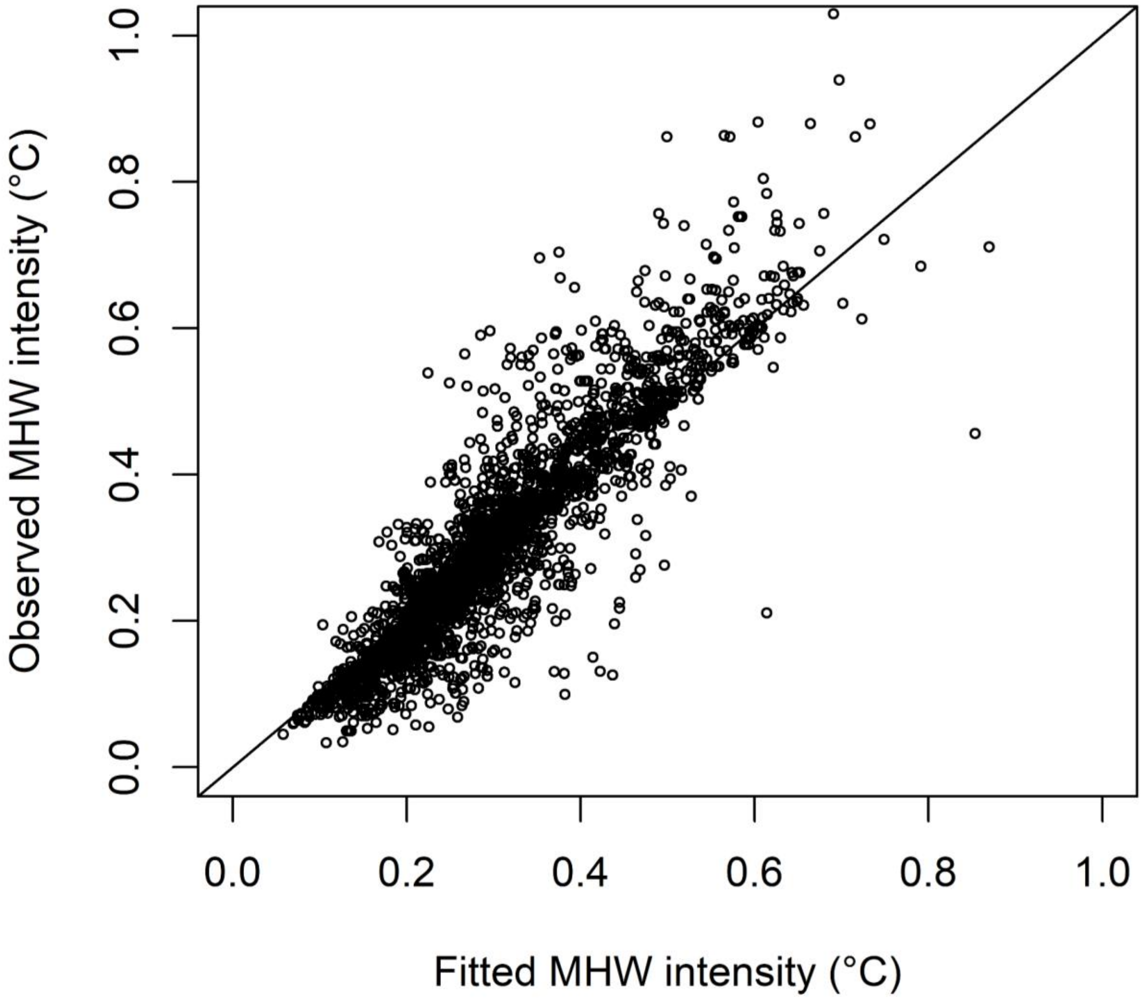

Supplementary Figure S8. The observed versus the fitted average intensity over the full duration of marine heatwaves from Equation 5. The observed data are mapped in Supplementary Figure S7.

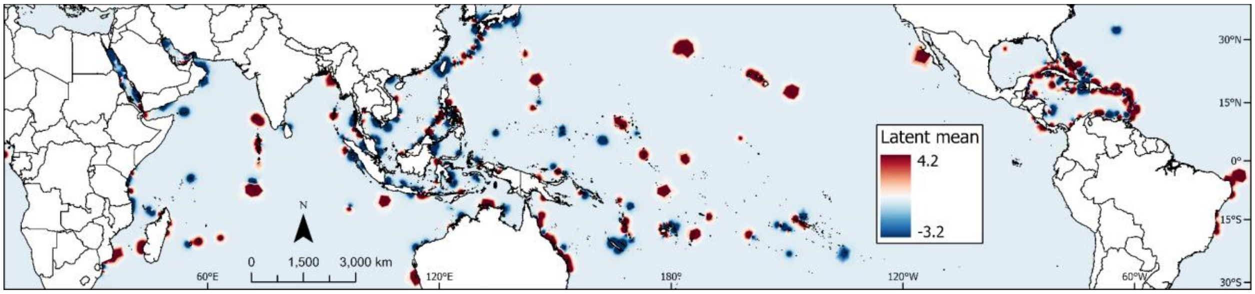

Supplementary Figure S9. The spatial-latent effect on the severity of coral bleaching for the world's shallow reefs (≤20 m) from 2002 to 2020. A positive spatial-latent effect (red) indicates where the observed coral bleaching was greater than expected by the model after accounting for the fixed effects, temporal effects, and the independent and identically distributed random effects, whereas a negative spatial-latent effect (blue) indicates where the observed coral bleaching was less than expected by the model after accounting for the fixed effects, temporal effects, and the independent and identically distributed random effects. This map is available as a raster file (see Data Availability). Map lines delineate study areas and do not necessarily depict accepted national boundaries.

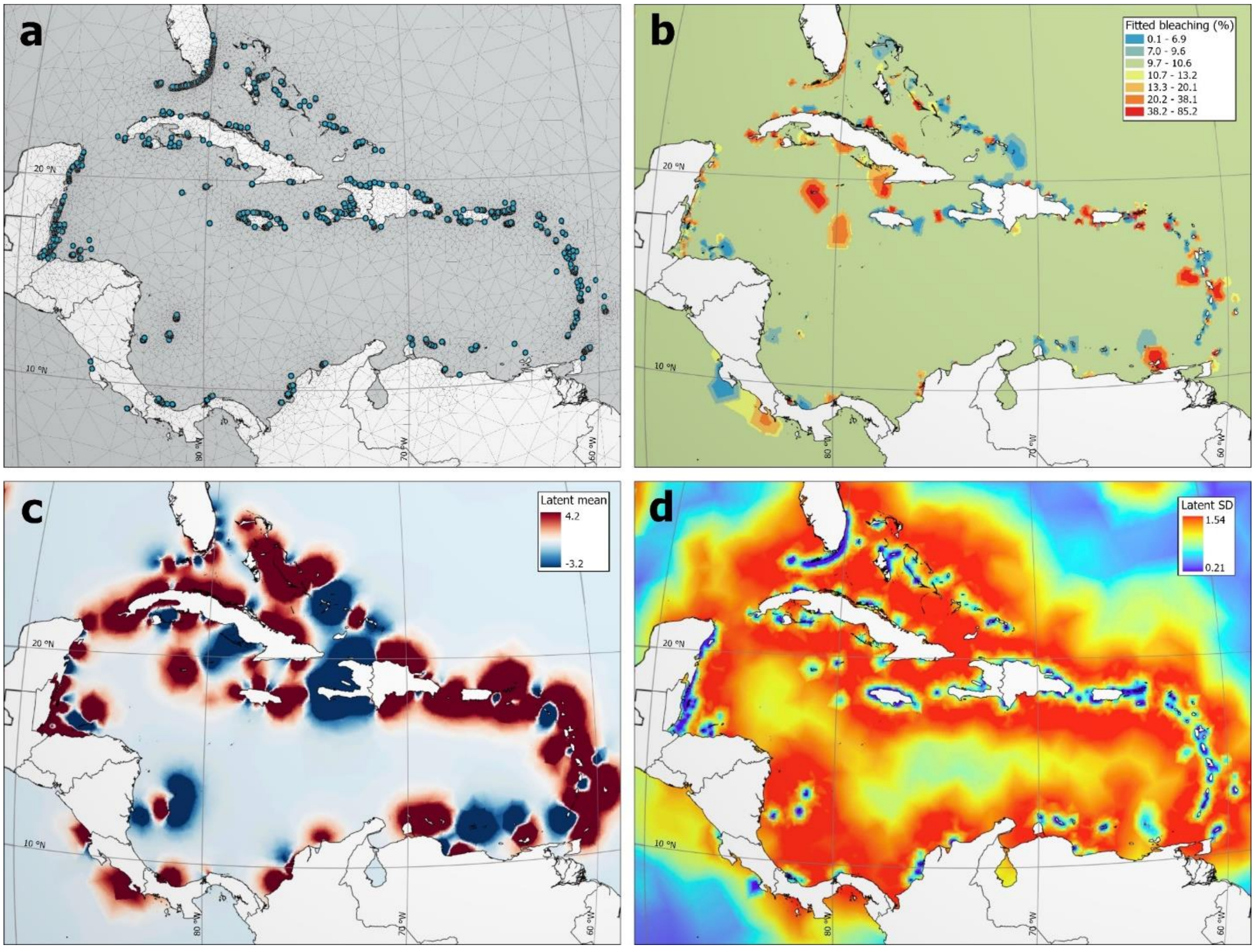

Supplementary Figure S10. *R-INLA* results for the Caribbean. (a) Discretized spherical Gaussian Markov random field (grey mesh) of the survey coordinates (blue circles), over which the stochastic partial differential equations were solved to approximate the spatial-latent effect. **(b)** Fitted coral bleaching revealed local-scale patchiness of bleaching severity. Blue areas indicate where the fitted coral bleaching was minimal, whereas red areas indicate where the fitted coral bleaching was moderate or severe. Sufficiently sampled areas have smooth, fine-scale (5 km) patterns (e.g., Florida, the Lesser Antilles, and the Yucatán Peninsula). Sparsely sampled areas have jagged, coarse-scale patterns (e.g., south of Cuba) because they received low triangulation when discretizing the Gaussian Markov random field as in **(a)**. Areas with no samples defaulted to the mean bleaching value. As the coral-bleaching data were extremely right-skewed, the color gradient is a seven-class geometric interval that allocates approximately the same number of pixels to each class. The fitted proportion of coral bleaching was converted back to a percentage. **(c)** The spatial-latent effect on the severity of coral bleaching captures geographical variation of coral bleaching unexplained by the fixed effects, temporal effects, and

the independent and identically distributed random effects. Dark blue represents where coral reefs bleached less than expected by the model after accounting for the fixed effects, temporal effects, and the independent and identically distributed random effects, whereas dark red represents where coral reefs bleached more than expected by the model after accounting for the fixed effects, temporal effects, and the independent and identically distributed random effects. The spatial-latent effect is on the logit scale and modeled at the 5-km resolution. **(d)** The standard deviation of latent coral bleaching indicates the precision of latent coral bleaching patterns, as in **(c)**. Blue represents where the mean latent effect is precise, whereas red represents where the mean latent effect is imprecise. Naturally, areas with sufficient sampling have a precise latent effect, whereas areas with sparse or no sampling have an imprecise latent effect, as in **(a)**. The standard deviation of the spatial-latent effect is on the logit scale and modeled at the 5-km resolution. Scale bars are not included because the images are perspective, top-down views of Earth from space, and perspective distortion causes the map scale to vary throughout the images, as indicated by the curved longitude and latitude graticules. North follows the longitude graticule upwards. Map lines delineate study areas and do not necessarily depict accepted national boundaries.

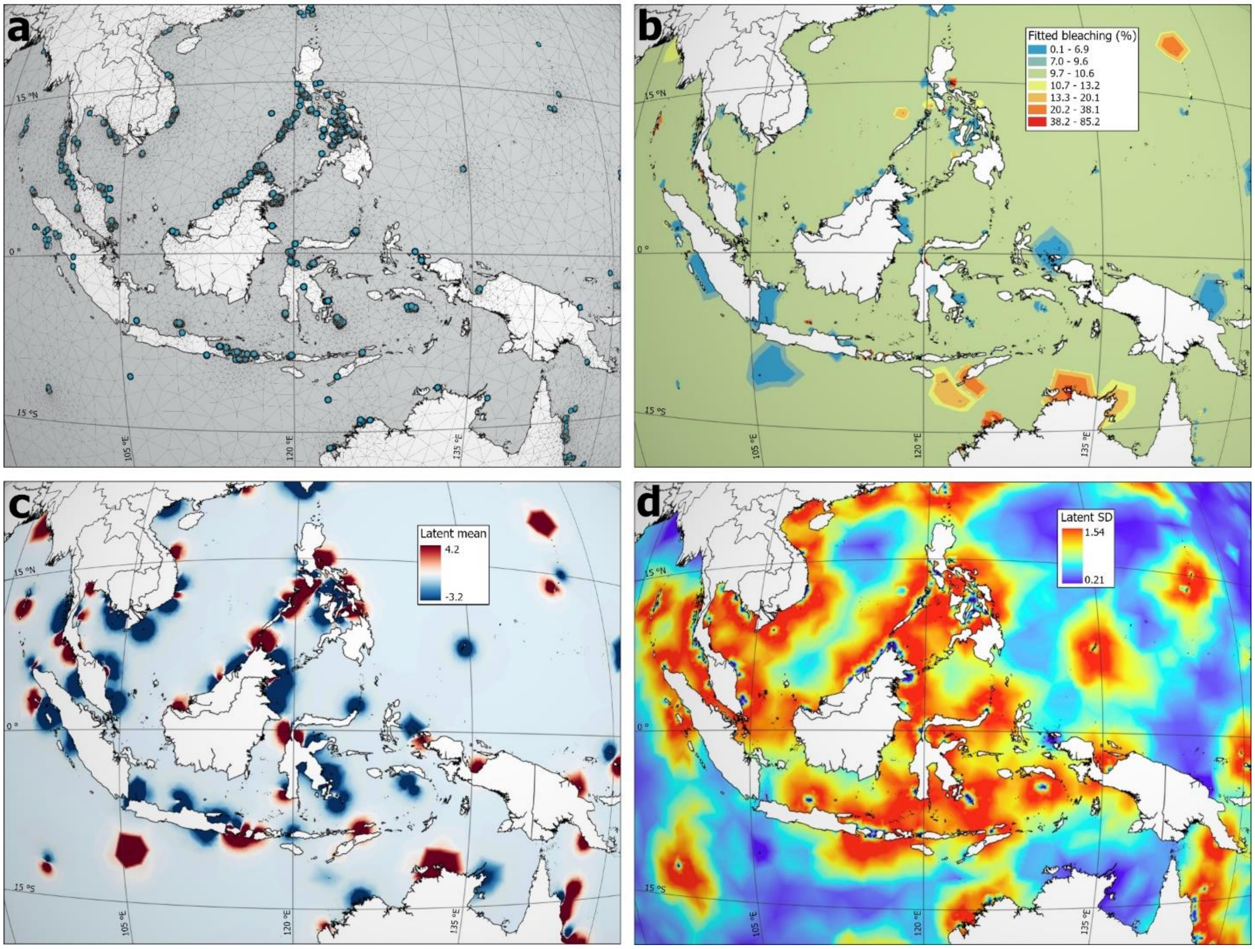

Supplementary Figure S11. ***R-INLA*** **results for the Coral Triangle. (a)** Discretized spherical Gaussian Markov random field (grey mesh) of the survey coordinates (blue circles), over which the stochastic partial differential equations were solved to approximate the spatial-latent effect. **(b)** Fitted coral bleaching revealed local-scale patchiness of bleaching severity. Blue areas indicate where the fitted coral bleaching was minimal, whereas red areas indicate where the fitted coral bleaching was moderate or severe. Sufficiently sampled areas have smooth, fine-scale (5 km) patterns. Sparsely sampled areas have jagged, coarse-scale patterns because they received low triangulation when discretizing the Gaussian Markov random field as in **(a)**. Areas with no samples defaulted to the mean bleaching value. As the coral-bleaching data were extremely right-skewed, the color gradient is a seven-class geometric interval that allocates roughly the same number of pixels to each class. The fitted proportion of coral bleaching was converted back to a percentage. **(c)** The spatial-latent effect on the severity of coral bleaching captures geographical variation of coral bleaching unexplained by the fixed effects, temporal effects, and the independent and identically distributed random effects. Dark blue represents where coral

reefs bleached less than expected by the model after accounting for the fixed effects, temporal effects, and the independent and identically distributed random effects, whereas dark red represents where coral reefs bleached more than expected by the model after accounting for the fixed effects, temporal effects, and the independent and identically distributed random effects. The spatial-latent effect is on the logit scale and modeled at the 5-km resolution. **(d)** The standard deviation of latent coral bleaching indicates the precision of latent coral bleaching patterns, as in **(c)**. Blue represents where the mean latent effect is precise, whereas red represents where the mean latent effect is imprecise. Naturally, areas with sufficient sampling have a precise latent effect, whereas areas with sparse or no sampling have an imprecise latent effect, as in **(a)**. The standard deviation of the spatial-latent effect is on the logit scale and modeled at the 5-km resolution. Scale bars are not included because the images are perspective, top-down views of Earth from space, and perspective distortion causes the map scale to vary throughout the images, as indicated by the curved longitude and latitude graticules. The distance between longitude graticules along the Equator is ~1,665 km. North follows the longitude graticule upwards. Map lines delineate study areas and do not necessarily depict accepted national boundaries.

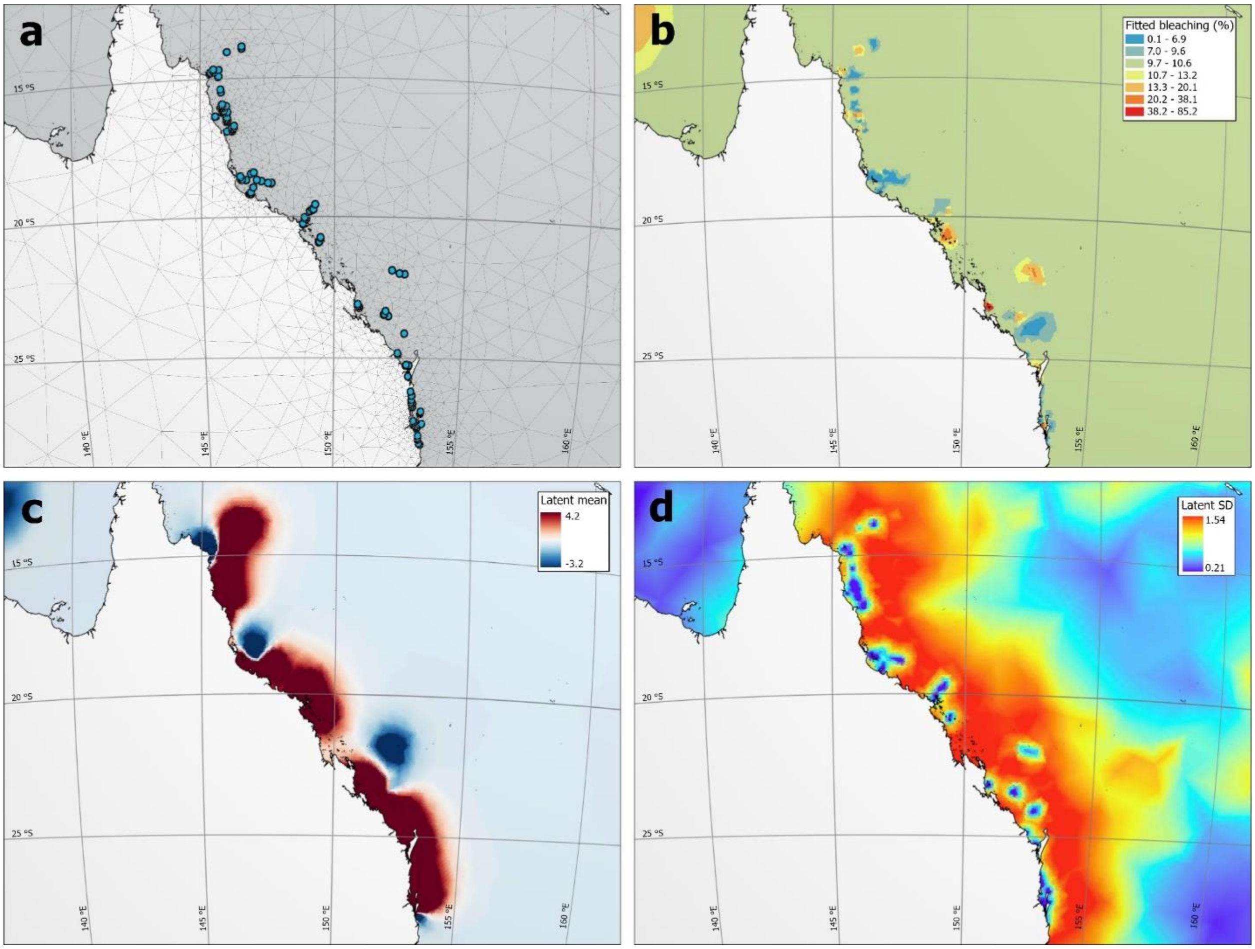

Supplementary Figure S12. *R-INLA* results for Australia's Great Barrier Reef. (a) Discretized spherical Gaussian Markov random field (grey mesh) of the survey coordinates (blue circles), over which the stochastic partial differential equations were solved to approximate the spatial-latent effect. **(b)** Fitted coral bleaching revealed local-scale patchiness of bleaching severity. Blue areas indicate where the fitted coral bleaching was minimal, whereas red areas indicate where the fitted coral bleaching was moderate or severe. Sufficiently sampled areas have smooth, fine-scale (5 km) patterns. Sparsely sampled areas have jagged, coarse-scale patterns because they received low triangulation when discretizing the Gaussian Markov random field as in **(a)**. Areas with no samples defaulted to the mean bleaching value. As the coral-bleaching data were extremely right-skewed, the color gradient is a seven-class geometric interval that allocates roughly the same number of pixels to each class. The fitted proportion of coral bleaching was converted back to a percentage. **(c)** The spatial-latent effect on the severity of coral bleaching captures geographical variation of coral bleaching unexplained by the fixed effects, temporal effects, and the independent and identically distributed random effects. Dark blue represents

where coral reefs bleached less than expected by the model after accounting for the fixed effects, temporal effects, and the independent and identically distributed random effects, whereas dark red represents where coral reefs bleached more than expected by the model after accounting for the fixed effects, temporal effects, and the independent and identically distributed random effects. The spatial-latent effect is on the logit scale and modeled at the 5-km resolution. **(d)** The standard deviation of latent coral bleaching indicates the precision of latent coral bleaching patterns, as in **(c)**. Blue represents where the mean latent effect is precise, whereas red represents where the mean latent effect is imprecise. Naturally, areas with sufficient sampling have a precise latent effect, whereas areas with sparse or no sampling have an imprecise latent effect, as in **(a)**. The standard deviation of the spatial-latent effect is on the logit scale and modeled at the 5-km resolution. Scale bars are not included because the images are perspective, top-down views of Earth from space, and perspective distortion causes the map scale to vary throughout the images, as indicated by the curved longitude and latitude graticules. North follows the longitude graticule upwards. Map lines delineate study areas and do not necessarily depict accepted national boundaries.

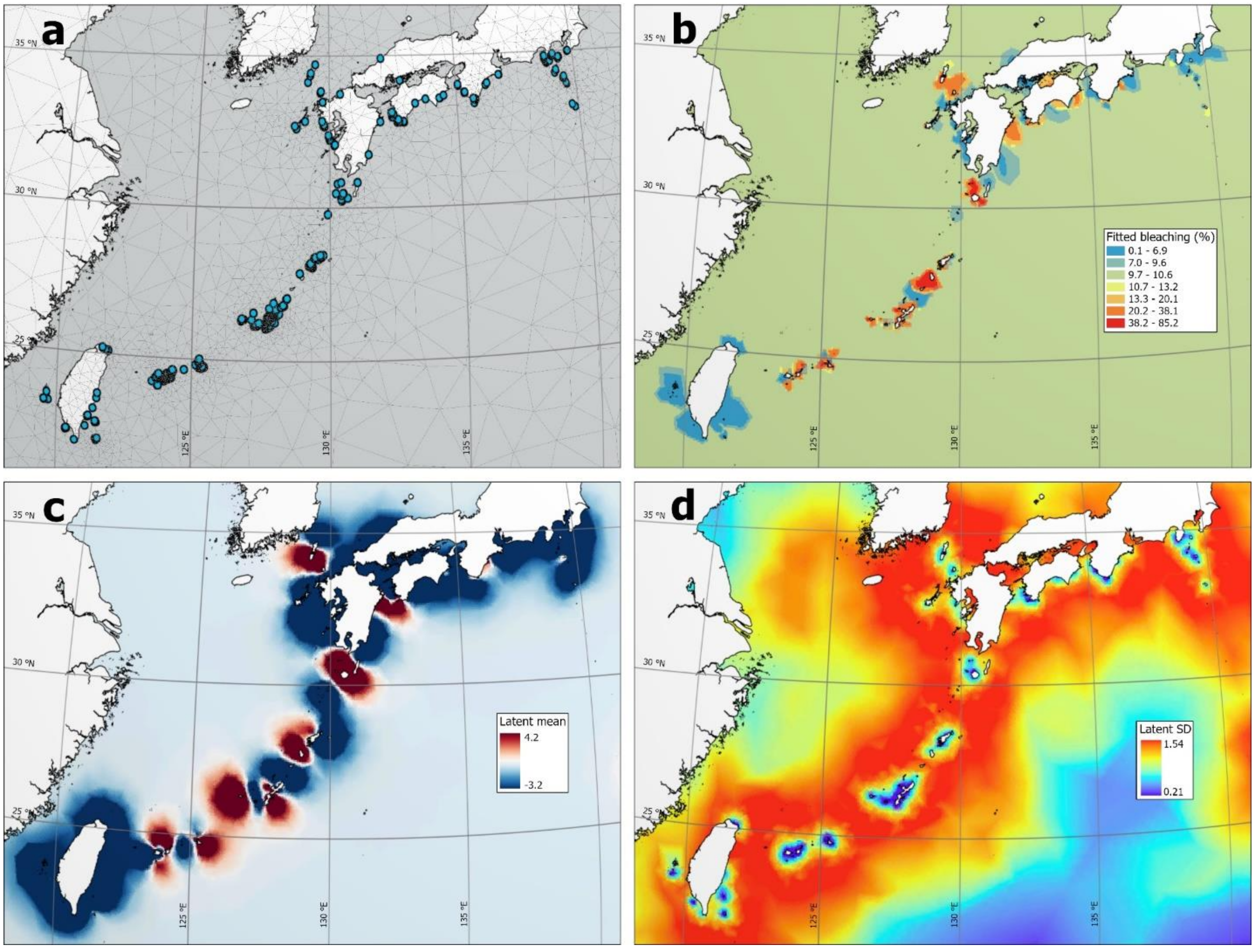

Supplementary Figure S13. ***R-INLA*** **results for Taiwan and Japan. (a)** Discretized spherical Gaussian Markov random field (grey mesh) of the survey coordinates (blue circles), over which the stochastic partial differential equations were solved to approximate the spatial-latent effect. **(b)** Fitted coral bleaching revealed local-scale patchiness of bleaching severity. Blue areas indicate where the fitted coral bleaching was minimal, whereas red areas indicate where the fitted coral bleaching was moderate or severe. Sufficiently sampled areas have smooth, fine-scale (5 km) patterns. Sparsely sampled areas have jagged, coarse-scale patterns because they received low triangulation when discretizing the Gaussian Markov random field as in **(a)**. Areas with no samples defaulted to the mean bleaching value. As the coral-bleaching data were extremely right-skewed, the color gradient is a seven-class geometric interval that allocates roughly the same number of pixels to each class. The fitted proportion of coral bleaching was converted back to a percentage. **(c)** The spatial-latent effect on the severity of coral bleaching captures geographical variation of coral bleaching unexplained by the fixed effects, temporal effects, and the independent and identically distributed random effects. Dark blue represents where coral

reefs bleached less than expected by the model after accounting for the fixed effects, temporal effects, and the independent and identically distributed random effects, whereas dark red represents where coral reefs bleached more than expected by the model after accounting for the fixed effects, temporal effects, and the independent and identically distributed random effects. The spatial-latent effect is on the logit scale and modeled at the 5-km resolution. **(d)** The standard deviation of latent coral bleaching indicates the precision of latent coral bleaching patterns, as in **(c)**. Blue represents where the mean latent effect is precise, whereas red represents where the mean latent effect is imprecise. Naturally, areas with sufficient sampling have a precise latent effect, whereas areas with sparse or no sampling have an imprecise latent effect, as in **(a)**. The standard deviation of the spatial-latent effect is on the logit scale and modeled at the 5-km resolution. Scale bars are not included because the images are perspective, top-down views of Earth from space, and perspective distortion causes the map scale to vary throughout the images, as indicated by the curved longitude and latitude graticules. North follows the longitude graticule upwards. Map lines delineate study areas and do not necessarily depict accepted national boundaries.

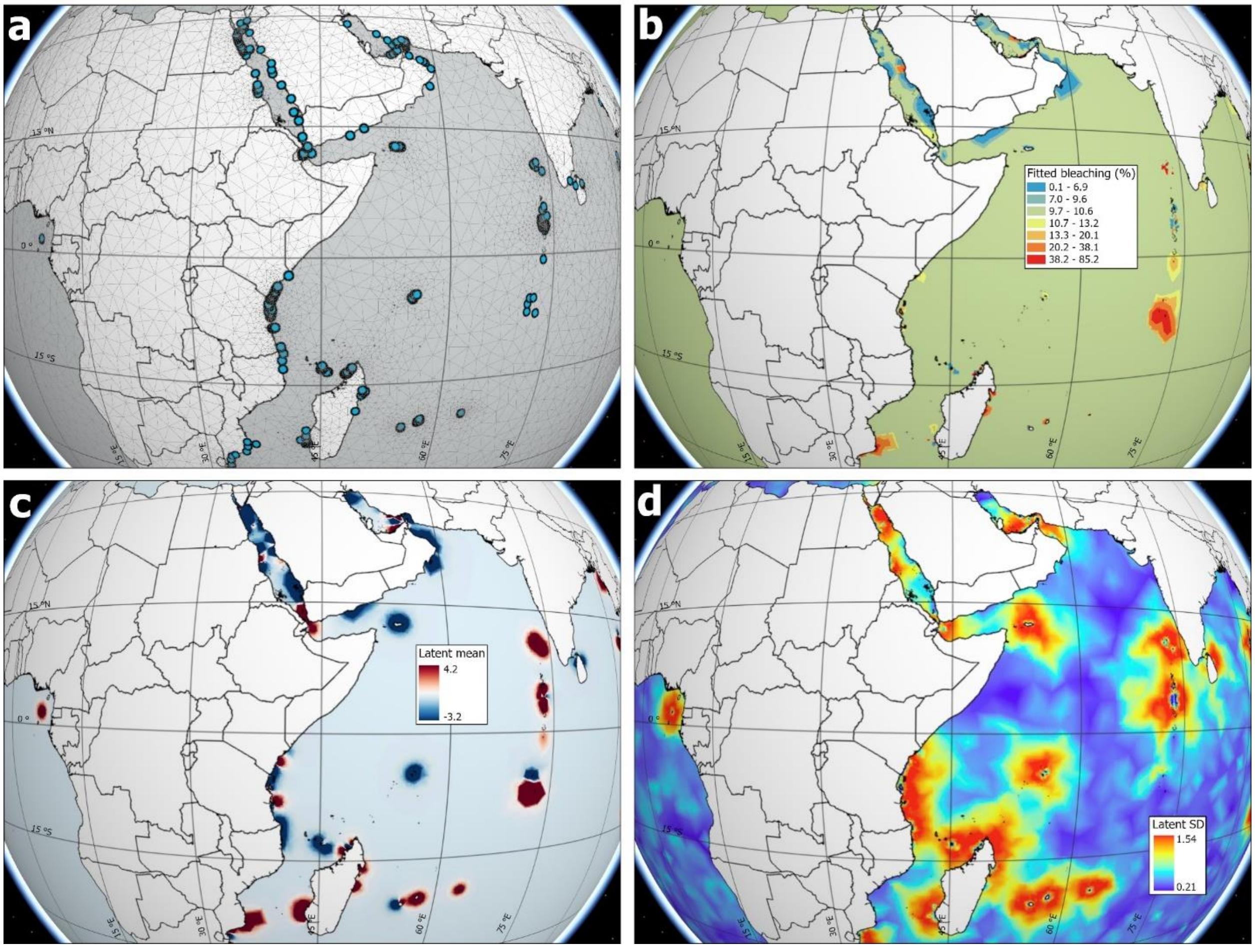

Supplementary Figure S14. *R-INLA* results for the western Indian Ocean. (a) Discretized spherical Gaussian Markov random field (grey mesh) of the survey coordinates (blue circles), over which the stochastic partial differential equations were solved to approximate the spatial-latent effect. **(b)** Fitted coral bleaching revealed local-scale patchiness of bleaching severity. Blue areas indicate where the fitted coral bleaching was minimal, whereas red areas indicate where the fitted coral bleaching was moderate or severe. Sufficiently sampled areas have smooth, fine-scale (5 km) patterns. Sparsely sampled areas have jagged, coarse-scale patterns because they received low triangulation when discretizing the Gaussian Markov random field as in **(a)**. Areas with no samples defaulted to the mean bleaching value. As the coral-bleaching data were extremely right-skewed, the color gradient is a seven-class geometric interval that allocates roughly the same number of pixels to each class. The fitted proportion of coral bleaching was converted back to a percentage. **(c)** The spatial-latent effect on the severity of coral bleaching captures geographical variation of coral bleaching unexplained by the fixed effects, temporal effects, and the independent and identically distributed random effects. Dark blue represents

where coral reefs bleached less than expected by the model after accounting for the fixed effects, temporal effects, and the independent and identically distributed random effects, whereas dark red represents where coral reefs bleached more than expected by the model after accounting for the fixed effects, temporal effects, and the independent and identically distributed random effects. The spatial-latent effect is on the logit scale and modeled at the 5-km resolution. **(d)** The standard deviation of latent coral bleaching indicates the precision of latent coral bleaching patterns, as in **(c)**. Blue represents where the mean latent effect is precise, whereas red represents where the mean latent effect is imprecise. Naturally, areas with sufficient sampling have a precise latent effect, whereas areas with sparse or no sampling have an imprecise latent effect, as in **(a)**. The standard deviation of the spatial-latent effect is on the logit scale and modeled at the 5-km resolution. Scale bars are not included because the images are perspective, top-down views of Earth from space, and perspective distortion causes the map scale to vary throughout the images, as indicated by the curved longitude and latitude graticules. The distance between longitude graticules along the Equator is ~1,665 km. North follows the longitude graticule upwards. Map lines delineate study areas and do not necessarily depict accepted national boundaries.

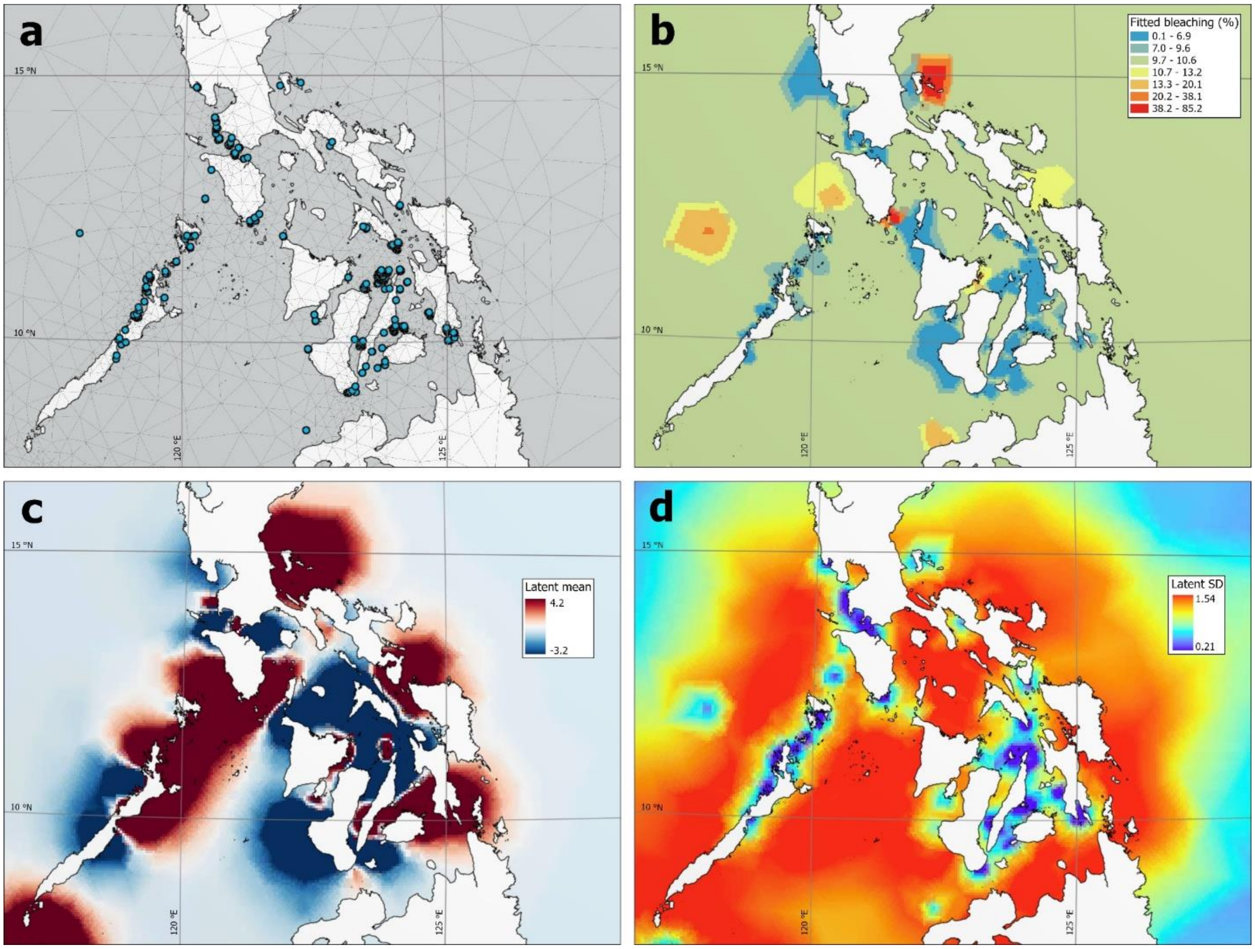

Supplementary Figure S15. *R-INLA* results for the Philippines. (a) Discretized spherical Gaussian Markov random field (grey mesh) of the survey coordinates (blue circles), over which the stochastic partial differential equations were solved to approximate the spatial-latent effect. **(b)** Fitted coral bleaching revealed local-scale patchiness of bleaching severity. Blue areas indicate where the fitted coral bleaching was minimal, whereas red areas indicate where the fitted coral bleaching was moderate or severe. Sufficiently sampled areas have smooth, fine-scale (5 km) patterns. Sparsely sampled areas have jagged, coarse-scale patterns because they received low triangulation when discretizing the Gaussian Markov random field as in **(a)**. Areas with no samples defaulted to the mean bleaching value. As the coral-bleaching data were extremely right-skewed, the color gradient is a seven-class geometric interval that allocates roughly the same number of pixels to each class. The fitted proportion of coral bleaching was converted back to a percentage. **(c)** The spatial-latent effect on the severity of coral bleaching captures geographical variation of coral bleaching unexplained by the fixed effects, temporal effects, and the independent and identically distributed random effects. Dark blue represents where coral

reefs bleached less than expected by the model after accounting for the fixed effects, temporal effects, and the independent and identically distributed random effects, whereas dark red represents where coral reefs bleached more than expected by the model after accounting for the fixed effects, temporal effects, and the independent and identically distributed random effects. The spatial-latent effect is on the logit scale and modeled at the 5-km resolution. **(d)** The standard deviation of latent coral bleaching indicates the precision of latent coral bleaching patterns, as in **(c)**. Blue represents where the mean latent effect is precise, whereas red represents where the mean latent effect is imprecise. Naturally, areas with sufficient sampling have a precise latent effect, whereas areas with sparse or no sampling have an imprecise latent effect, as in **(a)**. The standard deviation of the spatial-latent effect is on the logit scale and modeled at the 5-km resolution. Scale bars are not included because the images are perspective, top-down views of Earth from space, and perspective distortion causes the map scale to vary throughout the images, as indicated by the curved longitude and latitude graticules. North follows the longitude graticule upwards. Map lines delineate study areas and do not necessarily depict accepted national boundaries.

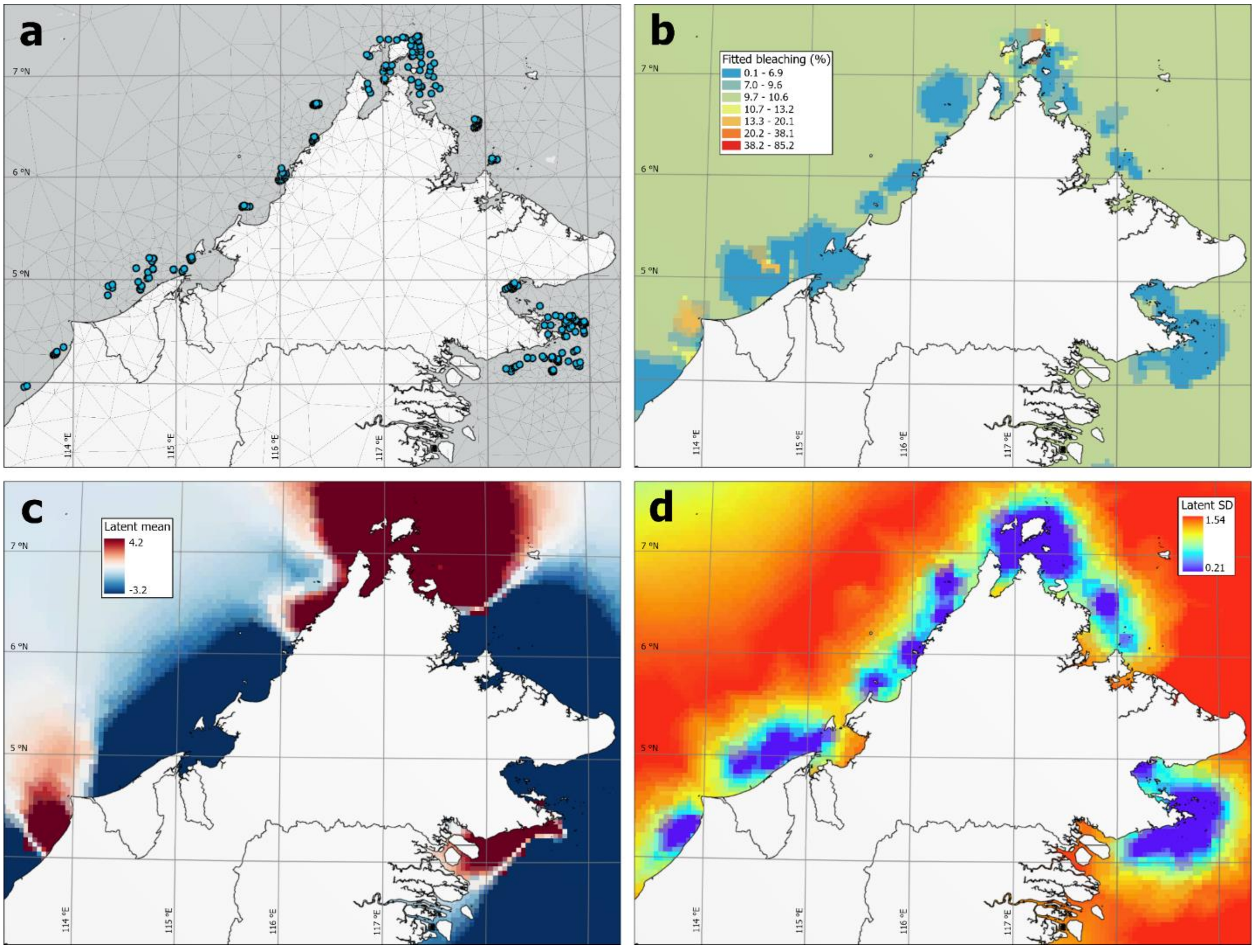

Supplementary Figure S16. ***R-INLA*** **results for northeast Borneo. (a)** Discretized spherical Gaussian Markov random field (grey mesh) of the survey coordinates (blue circles), over which the stochastic partial differential equations were solved to approximate the spatial-latent effect. **(b)** Fitted coral bleaching revealed local-scale patchiness of bleaching severity. Blue areas indicate where the fitted coral bleaching was minimal, whereas red areas indicate where the fitted coral bleaching was moderate or severe. Sufficiently sampled areas have smooth, fine-scale (5 km) patterns. Sparsely sampled areas have jagged, coarse-scale patterns because they received low triangulation when discretizing the Gaussian Markov random field as in **(a)**. Areas with no samples defaulted to the mean bleaching value. As the coral-bleaching data were extremely right-skewed, the color gradient is a seven-class geometric interval that allocates roughly the same number of pixels to each class. The fitted proportion of coral bleaching was converted back to a percentage. **(c)** The spatial-latent effect on the severity of coral bleaching captures geographical variation of coral bleaching unexplained by the fixed effects, temporal effects, and the independent and identically distributed random effects. Dark blue represents where coral

reefs bleached less than expected by the model after accounting for the fixed effects, temporal effects, and the independent and identically distributed random effects, whereas dark red represents where coral reefs bleached more than expected by the model after accounting for the fixed effects, temporal effects, and the independent and identically distributed random effects. The spatial-latent effect is on the logit scale and modeled at the 5-km resolution. **(d)** The standard deviation of latent coral bleaching indicates the precision of latent coral bleaching patterns, as in **(c)**. Blue represents where the mean latent effect is precise, whereas red represents where the mean latent effect is imprecise. Naturally, areas with sufficient sampling have a precise latent effect, whereas areas with sparse or no sampling have an imprecise latent effect, as in **(a)**. The standard deviation of the spatial-latent effect is on the logit scale and modeled at the 5-km resolution. Scale bars are not included because the images are perspective, top-down views of Earth from space, and perspective distortion causes the map scale to vary throughout the images, as indicated by the curved longitude and latitude graticules. North follows the longitude graticule upwards. Map lines delineate study areas and do not necessarily depict accepted national boundaries.

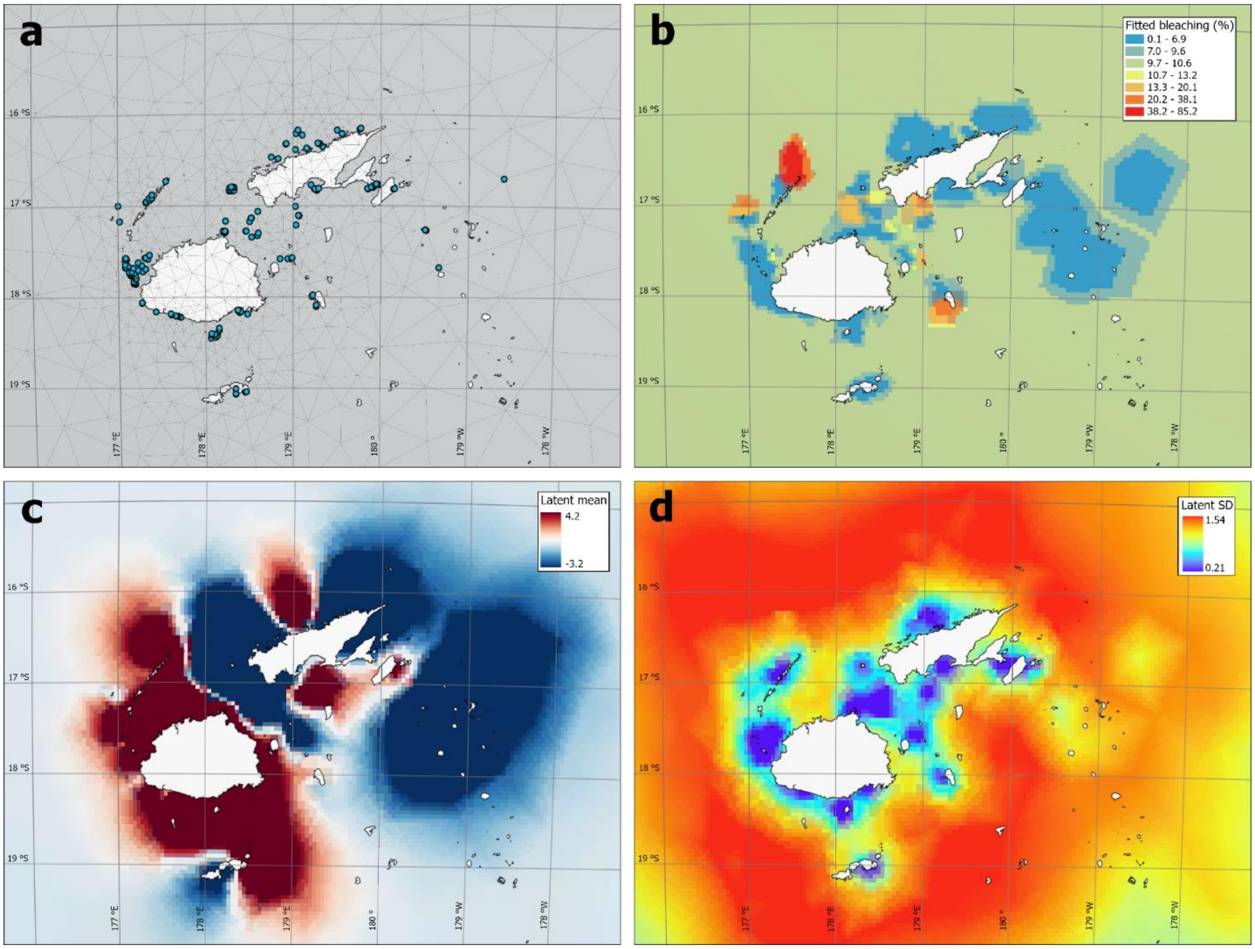

Supplementary Figure S17. ***R-INLA*** **results for Fiji. (a)** Discretized spherical Gaussian Markov random field (grey mesh) of the survey coordinates (blue circles), over which the stochastic partial differential equations were solved to approximate the spatial-latent effect. **(b)** Fitted coral bleaching revealed local-scale patchiness of bleaching severity. Blue areas indicate where the fitted coral bleaching was minimal, whereas red areas indicate where the fitted coral bleaching was moderate or severe. Sufficiently sampled areas have smooth, fine-scale (5 km) patterns. Sparsely sampled areas have jagged, coarse-scale patterns because they received low triangulation when discretizing the Gaussian Markov random field as in **(a)**. Areas with no samples defaulted to the mean bleaching value. As the coral-bleaching data were extremely right-skewed, the color gradient is a seven-class geometric interval that allocates roughly the same number of pixels to each class. The fitted proportion of coral bleaching was converted back to a percentage. **(c)** The spatial-latent effect on the severity of coral bleaching captures geographical variation of coral bleaching unexplained by the fixed effects, temporal effects, and the independent and identically distributed random effects. Dark blue represents where coral

reefs bleached less than expected by the model after accounting for the fixed effects, temporal effects, and the independent and identically distributed random effects, whereas dark red represents where coral reefs bleached more than expected by the model after accounting for the fixed effects, temporal effects, and the independent and identically distributed random effects. The spatial-latent effect is on the logit scale and modeled at the 5-km resolution. **(d)** The standard deviation of latent coral bleaching indicates the precision of latent coral bleaching patterns, as in **(c)**. Blue represents where the mean latent effect is precise, whereas red represents where the mean latent effect is imprecise. Naturally, areas with sufficient sampling have a precise latent effect, whereas areas with sparse or no sampling have an imprecise latent effect, as in **(a)**. The standard deviation of the spatial-latent effect is on the logit scale and modeled at the 5-km resolution. Scale bars are not included because the images are perspective, top-down views of Earth from space, and perspective distortion causes the map scale to vary throughout the images, as indicated by the curved longitude and latitude graticules. North follows the longitude graticule upwards. Map lines delineate study areas and do not necessarily depict accepted national boundaries.

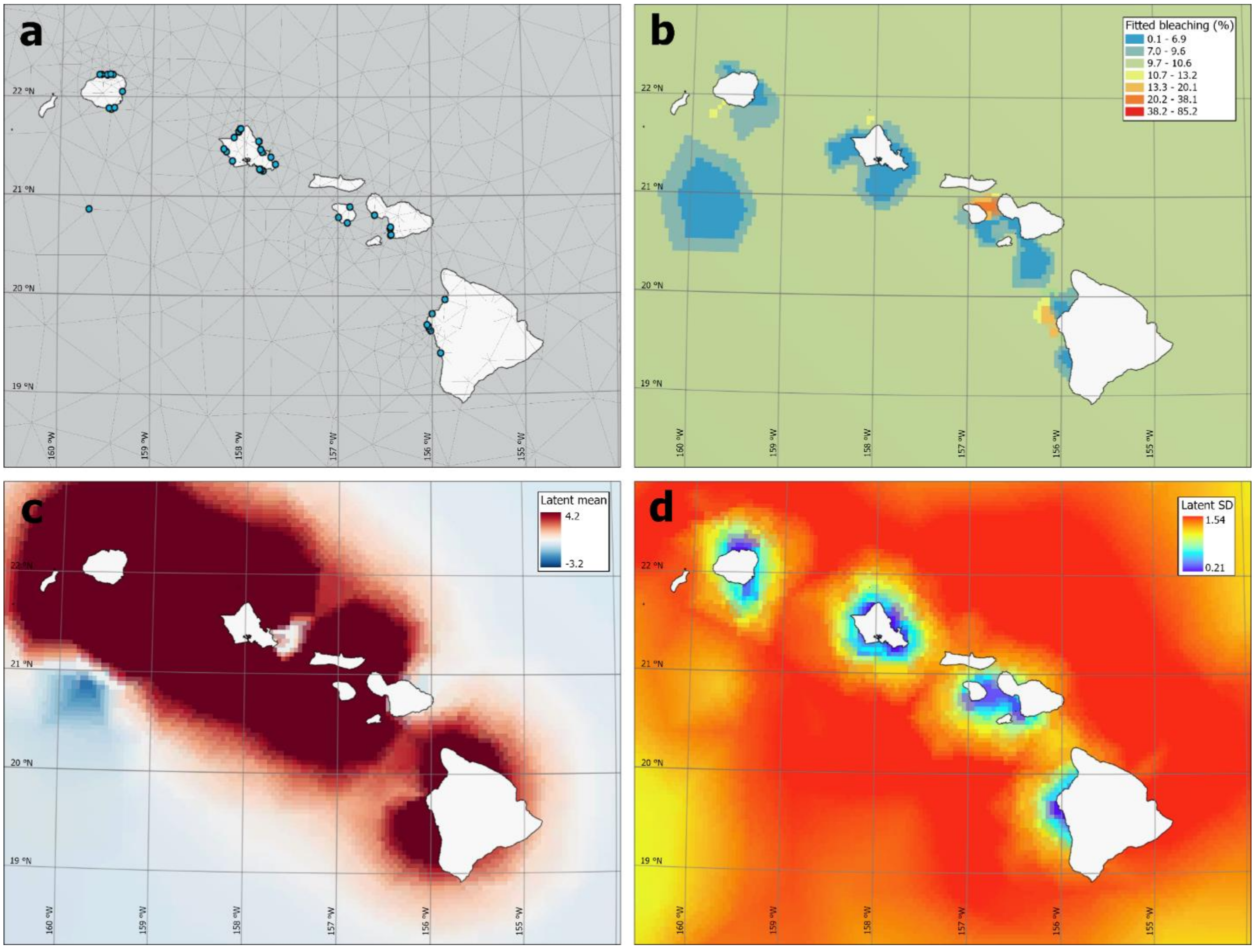

Supplementary Figure S18. ***R-INLA*** **results for the Main Hawaiian Islands. (a)** Discretized spherical Gaussian Markov random field (grey mesh) of the survey coordinates (blue circles), over which the stochastic partial differential equations were solved to approximate the spatial-latent effect. **(b)** Fitted coral bleaching revealed local-scale patchiness of bleaching severity. Blue areas indicate where the fitted coral bleaching was minimal, whereas red areas indicate where the fitted coral bleaching was moderate or severe. Sufficiently sampled areas have smooth, fine-scale (5 km) patterns. Sparsely sampled areas have jagged, coarse-scale patterns because they received low triangulation when discretizing the Gaussian Markov random field as in **(a)**. Areas with no samples defaulted to the mean bleaching value. As the coral-bleaching data were extremely right-skewed, the color gradient is a seven-class geometric interval that allocates roughly the same number of pixels to each class. The fitted proportion of coral bleaching was converted back to a percentage. **(c)** The spatial-latent effect on the severity of coral bleaching captures geographical variation of coral bleaching unexplained by the fixed effects, temporal effects, and the independent and identically distributed random effects. Dark blue represents

where coral reefs bleached less than expected by the model after accounting for the fixed effects, temporal effects, and the independent and identically distributed random effects, whereas dark red represents where coral reefs bleached more than expected by the model after accounting for the fixed effects, temporal effects, and the independent and identically distributed random effects. The spatial-latent effect is on the logit scale and modeled at the 5-km resolution. **(d)** The standard deviation of latent coral bleaching indicates the precision of latent coral bleaching patterns, as in **(c)**. Blue represents where the mean latent effect is precise, whereas red represents where the mean latent effect is imprecise. Naturally, areas with sufficient sampling have a precise latent effect, whereas areas with sparse or no sampling have an imprecise latent effect, as in **(a)**. The standard deviation of the spatial-latent effect is on the logit scale and modeled at the 5-km resolution. Scale bars are not included because the images are perspective, top-down views of Earth from space, and perspective distortion causes the map scale to vary throughout the images, as indicated by the curved longitude and latitude graticules. North follows the longitude graticule upwards. The ‘missing’ mesh over Hawai’i and Maui is a rendering bug. Map lines delineate study areas and do not necessarily depict accepted national boundaries.

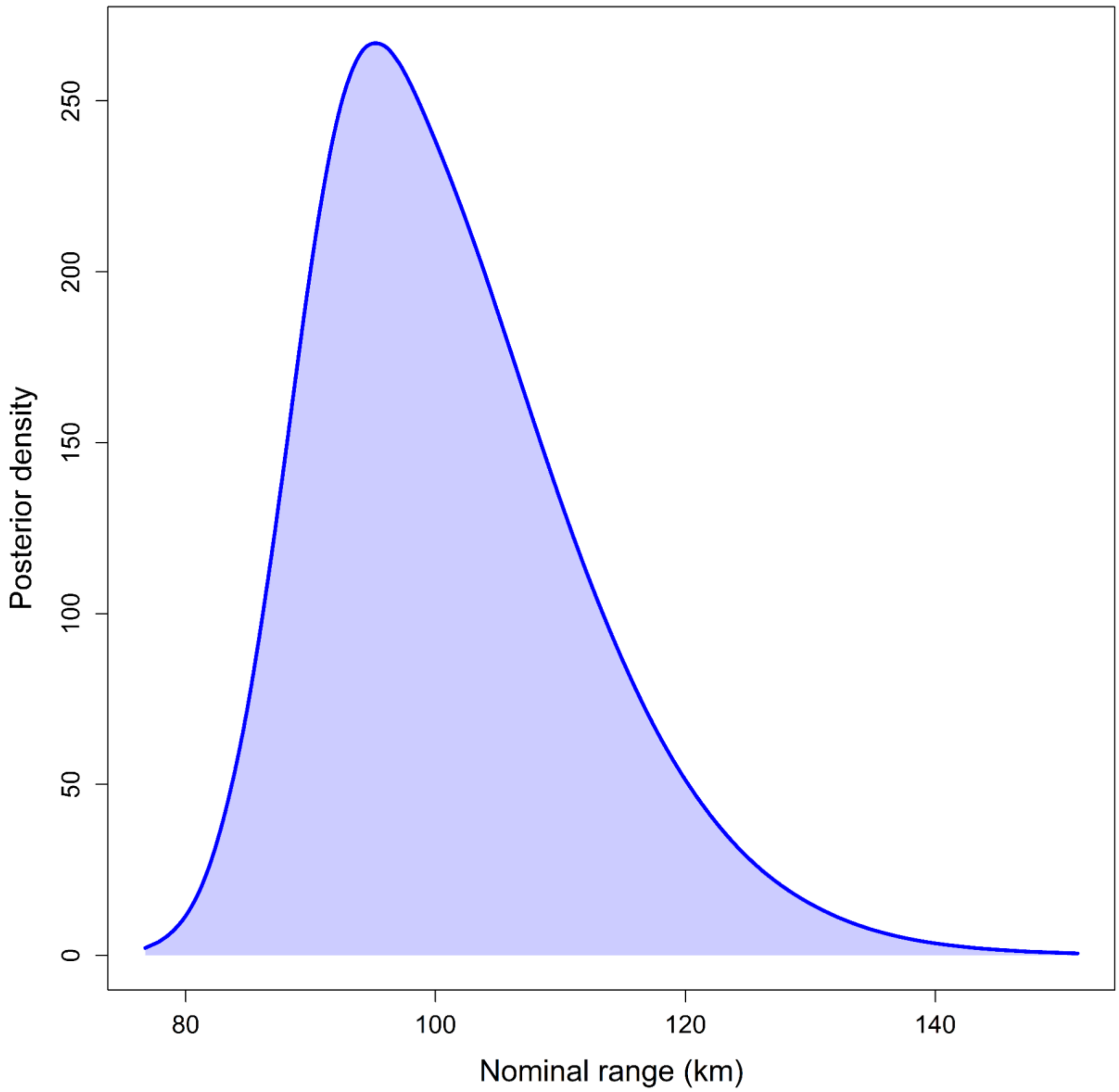

Supplementary Figure S19. Posterior distribution of the nominal range of coral bleaching from 30,266 coral-reef surveys at 8,728 sites (≤20 m) across 81 countries from 2002 to 2020. Here, the nominal range indicates the geodesic distance below which site-scale coral bleaching is spatially autocorrelated. The average nominal range is 101 km. Reefs that are separated by more than ~130 km are unlikely to have spatially autocorrelated site-scale coral bleaching during marine heatwaves.

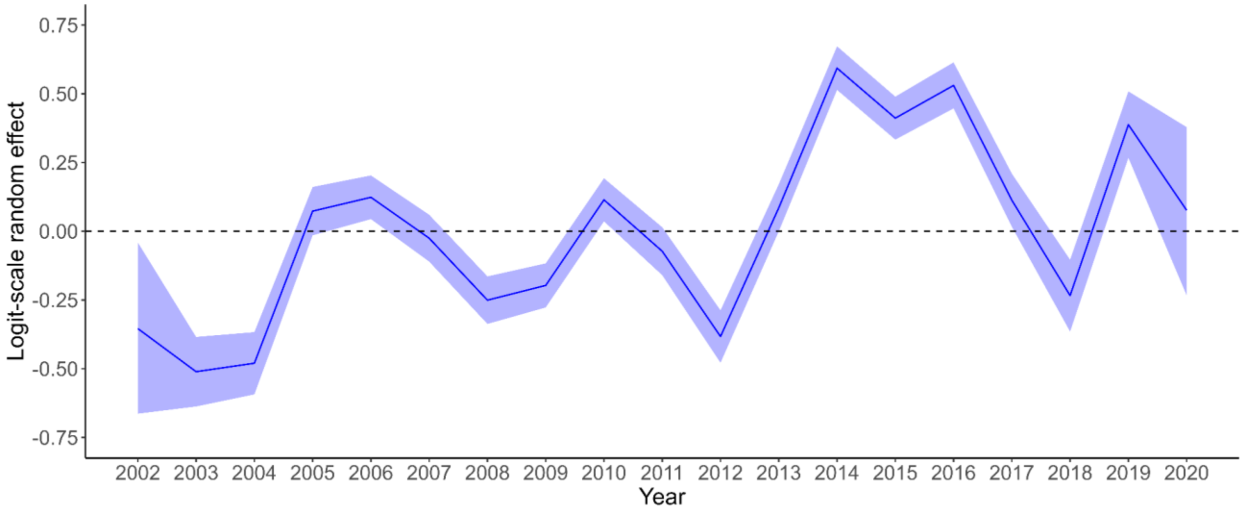

Supplementary Figure S20. Logit-scale random effect of the survey year on coral bleaching, from 30,266 coral-reef surveys at 8,728 sites (≤20 m) across 81 countries from 2002 to 2020, modeled as a first-order random walk in *R-INLA*. The solid line is the median, and the shading is the 95% credibility interval. Years where the random effect is positive increase the fitted log-odds of bleaching, whereas years where the random effect is negative decrease the fitted log-odds of bleaching.

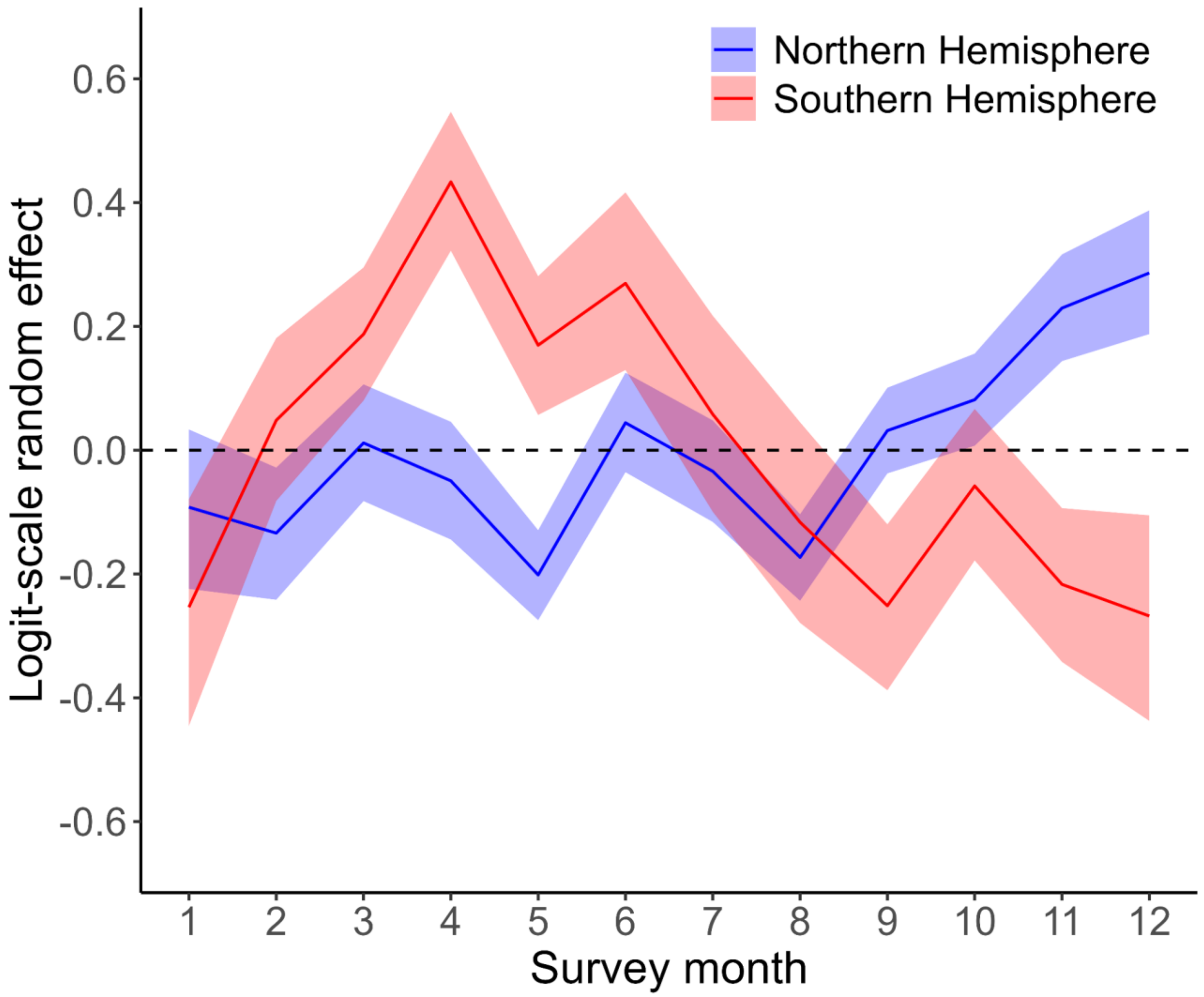

Supplementary Figure S21. Logit-scale random effect of the survey month on coral bleaching, from 30,266 coral-reef surveys at 8,728 sites (≤20 m) across 81 countries from 2002 to 2020, modeled as a cyclic first-order random walk in *R-INLA* for each hemisphere. The solid line is the median, and the shading is the 95% credibility interval. Months where the random effect is positive increase the fitted log-odds of bleaching, whereas months where the random effect is negative decrease the fitted log-odds of bleaching.

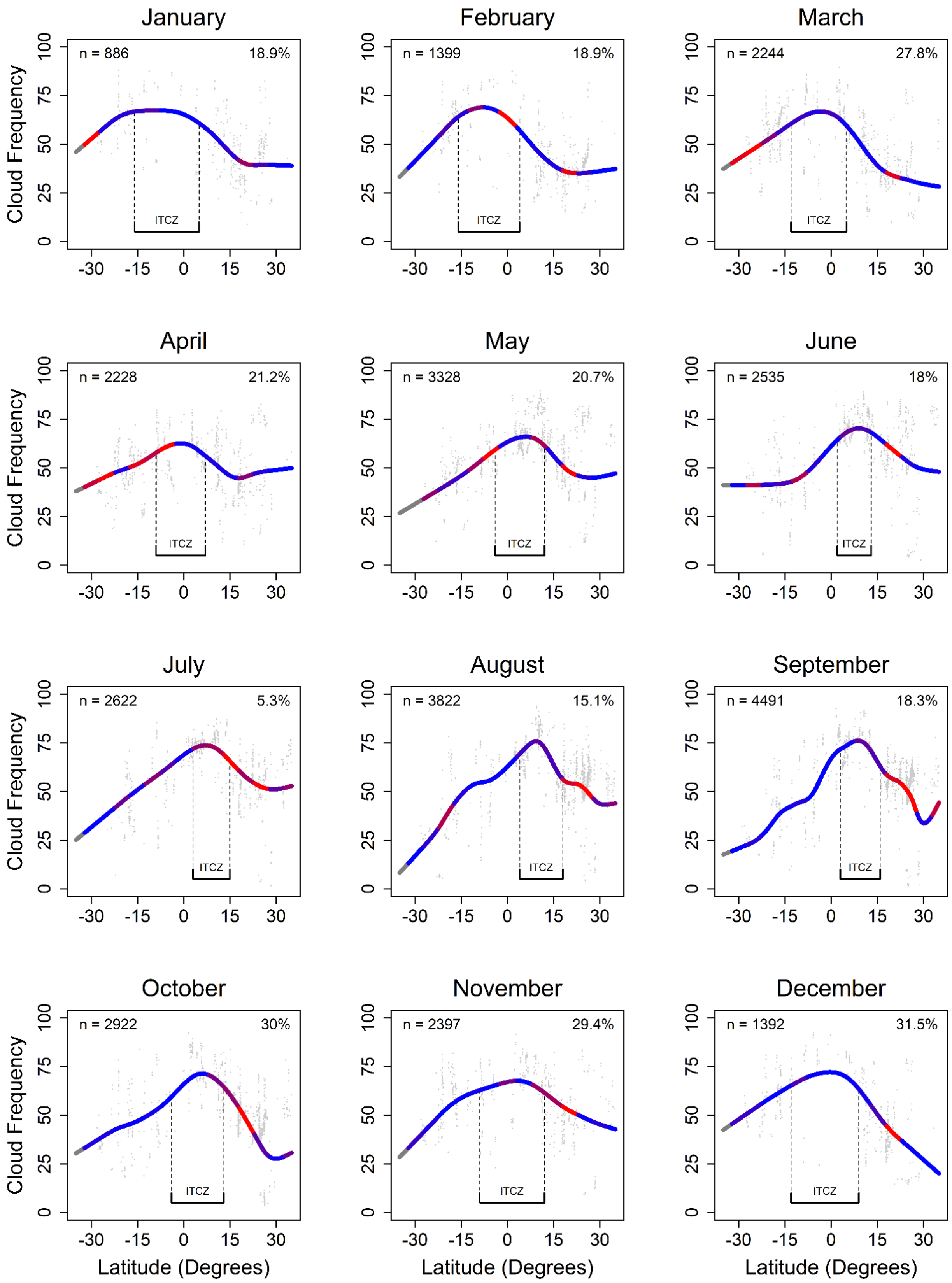
January
n = 886
18.9%
February
n = 1399
18.9%
March
n = 2244
27.8%
April
n = 2228
21.2%
May
n = 3328
20.7%
June
n = 2535
18%
July
n = 2622
5.3%
August
n = 3822
15.1%
September
n = 4491
18.3%
October
n = 2922
30%
November
n = 2397
29.4%
December
n = 1392
31.5%
ITCZ
Cloud Frequency
0
25
50
75
100
-30
-15
0
15
30
Latitude (Degrees)

Supplementary Figure S22. Monthly climatological cloud frequency (%), at 1-km resolution, at 8,728 coral-reef sites across 81 countries from 2000 to 2014. The climatological cloud frequency, which may moderate coral bleaching (Figure 3), is persistently highest near the Equator. The curve is a cubic spline of cloud frequency and latitude. The curve is colored by the average bleaching in latitude bins of 5 degrees, where blue is 0% bleaching, red is the maximum binned average percent bleaching across 2002–2020 for each month, which is provided at the upper right of each panel, and grey indicates insufficient samples for calculating average bleaching. The vertical dashed-black lines indicate the global-scale latitudinal range of the Intertropical Convergence Zone (ITCZ; Waliser & Gautier, 1993), which seasonally varies and facilitates cloud formation. The number of surveys (n) during each month is provided at the upper left of each panel.

Supplementary Table S1. Links to all the datasets we used. We cannot guarantee that all these links will be indefinitely accessible.

Variable	Dataset	Provider	Website
Coral bleaching and associated metadata	Global Coral-Bleaching Database	van Woesik & Kratochwill (2022)	https://www.bco-dmo.org/dataset/773466
Sea-surface temperature (5 km; daily)	CoralTemp	United States National Oceanic and Atmospheric Administration	https://www.star.nesdis.noaa.gov/pub/socd/mecb/crw/data/5km/v3.1_op/nc/v1.0/daily/sst/
Irradiance (4 km; daily)	Daily Photosynthetically Available Radiation	United States National Aeronautics and Space Administration	https://oceandata.sci.gsfc.nasa.gov/l3/
Turbidity (4 km; daily)	Daily Diffuse Attenuation Coefficient for Downwelling Irradiance at 490 nm	United States National Aeronautics and Space Administration	https://oceandata.sci.gsfc.nasa.gov/l3/
Current velocity (9.2 km; daily)	Global Ocean Physics Reanalysis	Copernicus Marine Service	https://data.marine.copernicus.eu/product/GLOBAL_MULTIYEAR_PHY_001_030/description
Significant wave height (22.2 km; 3-hourly)	Global Ocean Waves Reanalysis	Copernicus Marine Service	https://data.marine.copernicus.eu/product/GLOBAL_MULTIYEAR_WAV_001_032/description
Tidal range (3.7 km; 1992–2020 climatology)	Global Tidal Range (Finite Element Solution 2022)	Keith Van Graafeiland; AVISO+	https://www.arcgis.com/home/item.html?id=36eea2dda6194f66840ecb6f1af6ef0f
Cloud frequency (1 km; 2000–2014 climatology for each month)	Global 1-km Cloud Frequency	Wilson & Jetz (2016)	https://www.earthenv.org/cloud

Surface reflectance (10 m; daily)	Harmonized Sentinel-2 MultiSpectral Surface Reflectance	European Space Agency	https://developers.google.com/earth-engine/datasets/catalog/COPERNICUS_S2_SR_HARMONIZED
